# Real-time AI-driven trend analytics for smart city digital services using streaming data

**DOI:** 10.3389/fdata.2026.1811835

**Published:** 2026-08-14

**Authors:** Zhuldyz Kalpeyeva, Abdul Razaque, Raissa Uskenbayeva, Aliya Beishenaly, Venera Elle, Aizhan Kassymova

**Affiliations:** Department of Cybersecurity, Information Processing and Storage, Satbayev University, Almaty, Kazakhstan

**Keywords:** artificial intelligence, forecasting, real-time analytics, smart city, streaming data, trend detection

## Abstract

Real-time data analysis plays an important role in the operation of digital urban systems, where the behavior of residents and the load on services can change over short periods of time. At the same time, traditional analytical approaches based on batch data processing often do not allow timely detection of such changes, which leads to delayed and not always accurate management decisions. This paper introduces an artificial intelligence-driven smart city system (AISSC) for real-time data trend analysis. The proposed AISSC framework processes streaming data in a smart city digital environment. The proposed approach encompasses real-time feature generation and statistical techniques for identifying significant changes. The proposed AISSC solution is executed on the Python platform. The results demonstrate that the proposed AISSC solution achieves a directional accuracy of 98.6% for trend prediction, together with strong numerical forecasting performance with a MAPE of 6.3% and a WMAPE of 7.8%. The framework detects statistically significant deviations within 2.4 s at the sliding-window level while maintaining an end-to-end system update cycle of approximately 5 min. These results demonstrate the capability of the proposed framework to support reliable real-time trend analysis and short-term forecasting for smart city decision-making. This shows that the framework can reliably identify trends and accurately estimate demand for real-time smart city decision-making. The results demonstrate consistent performance improvements compared to representative baseline methods under identical streaming and computational constraints. The proposed AISSC facilitates real-time observation of urban dynamics and enhances short-term forecasting accuracy for informed decision-making in smart city management systems.

## 1 Introduction

The growing adoption of the digital economy and smart city models has amplified the significance of data in the administration of urban systems. Urban activities, which are inherently dynamic and exhibit spatiotemporal variability, demand data-driven decision-making to ensure the effective functioning of infrastructure (Liu et al., 2024). However, traditional batch-oriented systems encounter considerable difficulties in handling the magnitude and speed of the constantly generated urban data streams (Zhuang et al., 2024). This limitation leads to delayed decision-making, inefficient resource allocation, and missed opportunities in rapidly evolving urban environments. Therefore, the problem is particularly evident in smart cities, where data comes from a variety of diverse sources, including digital services, mobile applications, transportation systems, and sensor infrastructure (Razaque et al., 2025b). The use of aggregated reports with a large time step does not allow us to identify short-term behavioral changes and local anomalies of urban activity (Shetgaonkar et al., 2025). In addition, reliance on historical averages often hides dramatic shifts in the behavior of citizens, while outdated analytical solutions do not provide a comprehensive analysis of interrelated urban processes in real time (Xu et al., 2024; Ma et al., 2024). This limits the ability of urban systems to respond on time to changes in demand for services and infrastructure.

The limitations of traditional analytical approaches directly affect the efficiency of urban systems and the quality of services provided. In practice, this results in an overload of individual services, uneven use of infrastructure, and increased operating costs (Rojanasuvan, 2024; International Telecommunication Union, 2024). With insufficient adaptability of analytical tools, the user experience of citizens also deteriorates, since digital services do not have time to take into account the real needs of the population and changing behavioral patterns (Razaque et al., 2025a). Taken together, these factors reduce the overall effectiveness of urban environment management and require a review of existing analytical approaches. In order to overcome these limitations, these studies proposes a trend analysis system based on artificial intelligence methods, focused on the digital environment of a smart city (AlZahrani, 2026; Papastefanopoulos et al., 2023). The system is designed to process high-frequency data streams generated by urban platforms and services and can be used as part of the digital transformation processes of cities, including countries with developing digital infrastructure, such as Kazakhstan (Kumar et al., 2025; Davenport, 2018). The solution is based on a unified architecture that combines temporal granularity of data, statistical reliability, and practical applicability to support management decisions (Ogunkan and Ogunkan, 2025). The architecture includes three interrelated modules: streaming data processing and real-time feature generation, statistical trend detection and monitoring of changes in urban activity, as well as a short-term forecasting module using enriched analytical signals (Dobre et al., 2025). Another study focuses on three key issues: the impact of analytical processing delays on the effectiveness of urban system management; the role of real-time feature generation and statistical monitoring in reliable detection of urban trends; and the extent to which trend-based signals improve the accuracy of short-term predictive models in a smart city digital environment. By combining methods of streaming analytics, adaptive machine learning, and applied control logic, the proposed system forms a holistic approach to smart city data analysis that can be applied not only in a local context but also in other rapidly developing urban ecosystems that require prompt and informed decision support (Sanchez-Sepulveda et al., 2024). Unlike current streaming frameworks, which mainly focus on processing data, the proposed AISSC framework emphasizes integrated analytical intelligence and decision support within a single, real-time pipeline. The proposed framework exhibits notable performance enhancements, but its significance lies mostly in the system-level integration of several analytical components rather than in a solitary algorithmic progression.

### 1.1 Novelty and research contributions

The novelty of the proposed AISSC framework is innovative in three ways. Initially, the study introduces quantitative indicators for temporal granularity and cross-resource comparability at the theoretical level. Additionally, it offers a statistically grounded trend-detection formulation that is based on adaptive baselines and hypothesis testing. Secondly, the work integrates streaming feature engineering, trend-aware monitoring, short-term forecasting, and reinforcement-learning-based decision support into a unified end-to-end analytics pipeline at the integrative level, rather than considering these components as isolated modules. Third, the framework is specifically designed for smart city digital services, such as transportation, healthcare, and e-government environments, where low-latency analytics and adaptive operational response are essential from an applied perspective.

The novelty of this work is not the introduction of a fundamentally new forecasting algorithm. Instead, it is the design and implementation of an integrated end-to-end streaming analytics system that combines feature engineering, statistically sound trend detection, forecasting, and decision support into a single real-time framework.

Although the proposed AISSC framework emphasizes system-level integration, it also introduces several methodological advances beyond conventional smart city analytics architectures. First, the framework introduces quantitative measures of temporal granularity and cross-resource comparability that characterize streaming urban behavior across heterogeneous services. Second, AISSC employs a statistically grounded trend-detection mechanism that combines adaptive EWMA baselines with formal hypothesis testing, enabling the reliable separation of significant behavioral changes from random fluctuations in streaming environments. Third, the framework establishes a closed-loop analytical workflow in which trend-aware forecasting and reinforcement-learning-based decision support operate on continuously updated streaming representations. These methodological components extend existing architectures that primarily focus on data ingestion, visualization, or isolated prediction modules without integrating statistically validated trend intelligence and adaptive operational decision-making within a unified real-time framework. The proposed temporal granularity and cross-resource comparability measures are intended as analytical descriptors of streaming urban behavior rather than as standalone predictive performance metrics. Their theoretical properties are supported by the formal analysis presented in Hypothesis-1, Hypothesis-2, Lemma-1, and Corollary-1, which demonstrate that short-window aggregation preserves fine temporal variations while feature normalization improves stability and comparability across heterogeneous resources. The purpose of these metrics is to characterize the quality and consistency of streaming representations used by downstream analytical modules. A comprehensive benchmarking study against alternative temporal similarity or representation-quality measures is beyond the scope of the present work and constitutes an important direction for future research.

Unlike existing smart city analytics pipelines, where streaming analytics, trend analysis, forecasting, and operational decision support are typically implemented as separate or loosely coupled modules, the proposed AISSC framework integrates these components within a unified real-time analytical pipeline. The framework continuously transforms streaming events into normalized feature representations, performs statistically validated trend detection using adaptive EWMA baselines and hypothesis testing, enriches short-term forecasting with trend-aware signals, and employs reinforcement learning to support adaptive operational decisions. This tight integration enables a closed-loop analytics-to-decision workflow with low-latency operation, allowing urban services to react dynamically to evolving conditions. Therefore, the novelty of AISSC lies primarily in the system-level integration of statistically grounded trend intelligence, forecasting, and reinforcement-learning-based decision support within a single streaming architecture rather than in the introduction of an isolated prediction algorithm.

The main contributions are summarized as follows:

- The quantitative indicators are introduced that make it possible to assess the temporal detail and comparability of flow characteristics between different elements of the urban system. These indicators form a reliable basis for analyzing the quality and sustainability of the analytical pipeline and can be used in the design and evaluation of streaming analytics systems in smart cities.
- The statistically sound mechanism is developed for identifying trends, which integrates adaptive baseline data with formal hypothesis testing to make it possible to separate significant changes in urban activity from random fluctuations. This approach makes it possible to identify trends in a timely manner, reduces the number of false positives, and increases confidence in the results of the analysis among decision makers.
- A predictive module is developed that uses information about current changes and identified trends in the processing of streaming data. Unlike traditional approaches, which primarily depend on historical observations, the proposed module considers current signals reflecting the current state of urban activity. As new data arrives, forecasts can be updated, which improves their usefulness for short-term planning. As a result, an analytical system is being formed that is able to adapt to changes in the digital environment of a smart city and support more informed management decisions.

### 1.2 Problem identification and significance

Many urban digital systems still use analytical approaches based on batch data processing. Such systems lead to analysis delays of several hours or even days, which reduces their usefulness in a dynamic urban environment (Kadam and Kim, 2025). As a result, city authorities and service operators are often unable to respond in time to short-term load changes, local disruptions, or sudden risks that may arise within minutes or hours. In addition, existing decision support systems in smart cities are frequently oriented toward aggregated indicators and generalized performance metrics. With this approach, the possibility of detailed analysis of processes at the level of individual services, districts, or categories of urban activity in real time is lost. This makes it difficult to identify the causes of sudden changes in the use of urban services. The scale and complexity of data flows generated simultaneously by thousands of users, devices, and urban services impose fundamentally new requirements on the architecture of analytical systems. In this case, it is not enough to simply accelerate existing tools; it requires a transition to architectural solutions capable of continuously processing, analyzing, and transmitting information in accordance with the pace of change in urban activity.

The implementation of a real-time trend analysis system based on artificial intelligence methods allows for direct addressing of these limitations. By combining streaming data processing and machine learning algorithms, such a system provides an operational assessment of the current state of the urban environment, identification of short-term changes and support for adaptive management decisions. The adaptation of the analysis logic to the specifics of the functioning of urban services and infrastructure creates conditions for more flexible and informed management in the digital environment of a smart city.

### 1.3 Paper organization

The remainder of the article is organized as follows: Section 2 discusses the salient features of the existing work. Section 3 formulates the problem. Section 4 discusses the proposed solution. Section 5 describes the implementation and testing results. Section 6 discusses the results, including the limitations of the proposed approach. Section 7 concludes the entire article and provides future directions.

## 2 Related work

This section discusses the existing state-of-the-art methods. Cesario (2023) propose big data analytics-based urban management in smart cities. The suggested technique uses heterogeneous data sources, including IoT sensors, social media, and urban infrastructure systems, with machine learning and data mining to gain actionable insights. The framework monitors, predicts, and implements smart decisions for urban transportation, energy, and public services. The analysis of massive, high-velocity data streams improves urban efficiency and service quality. Multi-data integration enhances situational awareness and proactive management. Data privacy and security, scalability issues with growing data volumes, and data integration frameworks are all covered in the article. Good data and infrastructure may limit its use in developing digital ecosystems.

Lnenicka et al. (2024) examined the impact of big data analytics and data protection on the operations of 28 cities. The approach suggests that big data analytics can enhance urban services, optimize resource allocation, and facilitate evidence-based decision-making, all while integrating governance systems such as data protection, regulatory compliance, and risk management. This comprehensive, real-world analysis elucidates how cities navigate the interplay between data utilization and privacy, thereby fostering transparency, trust, and the advancement of smart city initiatives.

Consequently, large urban systems benefit from the accountability and data security provided by analytics guided by governance. The study also found differences in data governance policies, a lack of consistency in analytical frameworks, and difficulties in implementing data protection measures across different cities. Smart city systems, which use real-time analytics and artificial intelligence, often prioritize the technical aspects of their implementation over a thorough examination of the related policies.

Gubareva and Lopes (2024) suggested big data may aid smart city resource management. Big data analytics frameworks utilize large, heterogeneous urban datasets to facilitate the monitoring, optimization, and planning of urban resources, including transportation, energy, and public services. The identification of patterns through data mining, machine learning, and statistical analysis informs strategic decision-making within smart cities. This research's thorough examination and its applicability across diverse urban environments demonstrate how big data can improve resource allocation and operational effectiveness. Furthermore, they examined the scalability and maintenance of analytics within urban environments. However, the study is constrained by a generalized approach, lacking real-time system implementation, streaming data capabilities, low-latency analytics, and performance evaluation of deployments. The practical application of these solutions in dynamic smart cities may be impeded by challenges related to data integration, interoperability, and infrastructure readiness.

Kharlamov and Pilgun (2024) forecasted smart city growth using quantitative data and user perception. We research urban dynamics using data-driven models and incorporate public comments and behavioral insights to increase prediction accuracy and context. The recommended statistical analysis and machine learning method predict urban events, service demand, and environmental variables. This study analyzes urban systems and improves projections for real-world decision-making using factual data and subjective user judgments. Early emergency detection aids proactive management in complex cities. User-generated data may be biased, large-scale real-time streaming systems lack scalability, and the study does not interact with continuous, high-frequency data pipelines. Perception-driven analysis rather than automated real-time analytics may limit its use in latency-sensitive smart city applications.

Bafail (2024) enhanced smart city methods with random forest and regression. The proposed method assesses municipal performance and enhances strategic planning and decision-making processes through the utilization of urban data. Predictive modeling of smart city indicators, leveraging data, aims to enhance municipal operations through the analysis of complex interactions among various urban variables. Ensemble learning methodologies improve predictive accuracy and policy development by effectively addressing the nonlinear patterns present in urban datasets. Moreover, interpretable regression enhances the transparency of decision-making processes. Conversely, the study's applicability within dynamic urban settings is limited by its dependence on static or historical data.

The framework also lacks continuous data input, integration with real-time decision support systems, and low-latency processing capabilities. These constraints may limit its usage in smart city applications requiring rapid response and adaptive management. Bozkurt et al. (2025) proposed an urban data governance reference model for smart city data administration and utilization. Data quality, security, and adherence to regulations are ensured through governance structures that dictate data management rules, methods, and responsibilities. Municipalities leverage governance frameworks and data lifecycle management to facilitate data access, sharing, and utilization, thereby enabling data-informed decision-making. Consequently, governance enhances the transparency and accountability of urban data systems, while also bolstering confidence in smart city analytics. Moreover, the standardization of urban data governance methods helps cities use them effectively. Its conceptual and framework-based approach, without execution, limits the investigation. However, the study does not address real-time analytics integration, high-velocity streaming data, or dynamic scalability. The paradigm improves governance but requires technology integration for real-time AI-driven smart city analytics.

A data-driven smart city digital transformation architecture using AI, IoT readiness, and interactive dashboards is proposed by Lloret et al. (2025). AI-based analytics and data heterogeneity provide real-time urban surveillance, visualization, and decision support. Dashboards let stakeholders act on IoT data, and analytical modules evaluate and predict. Situational awareness and smart city management decisions are improved by data, analytics, and visualization. Decision-makers can see real-time data and analytics on dashboards. High-frequency data scalability and low-latency streaming are limited by the study. System integration and visualization trump advanced real-time predictive modeling and adaptive learning. In fast-paced, real-time smart cities, continuous analytics and enhanced AI-powered forecasting could boost their value.

Karampakakis et al. (2025) introduced a web-based application for smart city data analysis and visualization. It offers an engaging approach to urban data exploration. This proposed web application facilitates the real-time monitoring of smart city indicators and urban dynamics through the collection, analysis, and visualization of pertinent data. The application's design prioritizes usability, enabling both city administrators and citizens to leverage data-driven insights without necessitating specialized computer skills. Furthermore, web architecture provides remote access and data source integration, which in turn enhances both flexibility and scalability. However, the study's scope does not encompass advanced predictive analytics or real-time streaming data processing. The focus on visualization and human interaction over deep analytical modeling and automated decision-making may limit its effectiveness in highly dynamic smart city scenarios. Despite recent advances, significant gaps remain in smart city data analytics research.

Current investigations into smart city analytics are primarily characterized by batch-based models, static data processing, or discrete solutions centered on visualization, governance, or predictive capabilities, frequently lacking genuine real-time functionality. While recent research has initiated exploration into streaming-based IoT analytics and edge intelligence frameworks, these methodologies predominantly prioritize infrastructure-level optimizations, including data ingestion and low-latency processing, rather than integrated analytical intelligence. A significant constraint of current research lies in its inadequate capacity to analyze high-frequency streaming data, which is essential for capturing transient urban dynamics. While many methods use incremental processing or window-based aggregation, they often lack statistically sound ways to distinguish important behavioral patterns from random variations. Moreover, forecasting, anomaly detection, and decision support are often treated as separate tasks. This separation leads to fragmented analytical processes instead of the creation of integrated, real-time systems.

Another significant issue is the limited use of adaptive learning methods. While some strategies incorporate online learning or basic predictive models, they rarely combine streaming feature engineering with decision optimization driven by reinforcement learning, especially under conditions of strict latency constraints. Furthermore, discussions of scalability frequently focus on system throughput, neglecting to sufficiently consider the maintenance of analytical precision and responsiveness amidst escalating data velocity and heterogeneity.

Consequently, a comprehensive framework is evidently required to integrate streaming data processing with dependable trend detection, real-time forecasting capabilities, and flexible decision-making support systems. The proposed AISSC framework is designed to address several of these limitations by incorporating hypothesis-driven trend modeling, streaming feature engineering, and reinforcement learning within a cohesive, low-latency, and scalable architecture, specifically engineered to accommodate the dynamic characteristics of smart city environments. Table 1 shows a comparative analysis of existing methods. The comparison reflects both algorithmic capabilities and system-level integration characteristics. The qualitative thresholds (Low, Medium, High) are assigned based on the defined quantitative levels: < 70% signifies low, 70%−90% denotes moderate, and > 90% represents high.

**Table 1:** Comparative analysis of existing research in real-time smart city analytics.

Methods	Application domain	Data processing mode	Core analytical technique	Trend detection capability	Forecasting integration	Latency awareness	Scalability consideration	Decision support readiness	Key limitations
Cesario (2023)	Urban management (transport, energy, services)	Mixed (batch + partial streaming)	ML + data mining on heterogeneous data	Implicit (pattern discovery, not formalized)	Yes (predictive analytics)	Not explicitly defined	Scales with big data infrastructure (requires high resources)	Partial (insight-driven, not real-time)	Data privacy issues, heavy infrastructure dependency, and no low-latency pipeline
Lnenicka et al. (2024)	Urban governance and data protection	Batch/analytical evaluation	Policy-driven analytics + governance models	None	No	No	Limited (policy-focused, not system-level scaling)	Indirect (governance-level decisions)	No real-time analytics; lack of a unified analytical framework
Gubareva and Lopes (2024)	Resource management in smart cities	Batch-oriented big data	Data mining + statistical analysis	Implicit (pattern-level)	Yes (strategic planning)	No	Moderate (conceptual scalability only)	Low (not operational)	No streaming support; no latency awareness; no deployment evaluation
Kharlamov and Pilgun (2024)	Urban forecasting with user perception	Batch/hybrid	ML + statistical models + user feedback	Partial (perception-driven trends)	Yes	Not defined	Limited for high-frequency data	Medium (decision insights)	Bias in user data; no real-time streaming; weak scalability
Bafail (2024)	Urban performance prediction	Batch/historical	Random Forest + Regression	None	Yes	No	Moderate (model-level scaling)	Medium (policy support)	Static data dependency; no real-time capability; no adaptive system
Bozkurt et al. (2025)	Smart city data governance	Conceptual/ framework-based	Governance model	None	No	No	Not applicable (no system implementation)	Low	No implementation; no analytics integration; no streaming capability
Lloret et al. (2025)	Smart city dashboards and AI analytics	Near real-time (dashboard-level)	AI analytics + visualization	Limited (visual trend observation)	Yes	Partial (UI-level responsiveness)	Moderate (dashboard scaling)	Medium (human-in-loop)	Focus on visualization; lacks deep predictive modeling; limited streaming
Karampakakis et al. (2025)	Web-based smart city analytics	Near real-time (web app)	Data analytics + visualization	None	No	Partial (UI responsiveness only)	Moderate	Low–medium	No predictive analytics; no automated decision-making; weak real-time processing
**Proposed AISSC framework**	Smart city digital services	Streaming (high-frequency, sliding windows)	EWMA + statistical testing + ML + RL	Explicit (statistically validated, hypothesis-based)	Yes (real-time enriched forecasting)	Yes (~2.4 s detection latency)	High (distributed, modular architecture)	Conceptual (supports decision-making through integrated analytics; practical deployment)	Parameter sensitivity under extreme volatility

## 3 Problem formulation

Smart city settings produce high-frequency, diverse event streams stemming from transportation networks, digital government services, healthcare provisions, and IoT architectures. This research endeavors to create a real-time analytical framework designed to discern statistically significant patterns and predict short-term demand, all while adhering to stringent limitations regarding latency, scalability, and volatility. The system processes continuous data streams to (i) extract temporally granular features, (ii) identify deviations from adaptive baselines, and (iii) facilitate predictive and decision-making functions.

The incoming urban stream *E* is modeled as continuous, ordered temporal sequence of events generated by smart city services and infrastructure elements. The incoming urban data stream *E* is defined in Equation 1.


$$\begin{array}{l} E={\left(e_{k}\right)}_{k=1}^{\infty },e_{k}=\left(u_{k},i_{k},a_{k},c_{k},t_{k}\right) \end{array}$$ (1)

Each event in the stream corresponds to a user interaction or operational activity on a particular smart city resource or service. The tuple fields correspond respectively to the event originator, the resource identifier it is associated with, the action type, the contextual feature vector, and the event timestamp.

where *u*_*k*_ denotes the user generating event, *i*_*k*_ is the associated resource, *a*_*k*_ denotes the action type, *c*_*k*_ represents the contextual feature vector, and *t*_*k*_ indicates the event timestamp.

The resource identifier associates each event with a unique operational entity monitored within the smart city environment.

In this study, the term resource consistently refers to an individual operational entity within the smart city ecosystem, such as transportation route, healthcare facility, digital government service, or IoT-enabled infrastructure component.

For each resource *i*_*k*_ and current time *t*, a sliding time window $W_{i}\left(t\right)$ is defined in Equation 2.


$$\begin{array}{l} W_{i}(t)=\{e_{k}\in ℰ|r_{k}=i,\;t-\Delta \leq t_{k}\leq t\} \end{array}$$ (2)

This window captures short-term dynamics essential for real-time analytics.

### 3.1 Event-based metric computation

Within each window, three primary activity metrics are computed. The count of entry events is determined in Equation 3.


$$\begin{array}{l} E_{i}\left(t\right)=\underset{e_{k}\in W_{i}\left(t\right)}{\sum }𝕀\left(a_{k}=\text{entry}\right) \end{array}$$ (3)

The count of request events is given in Equation 4.


$$\begin{array}{l} R_{i}\left(t\right)=\underset{e_{k}\in W_{i}\left(t\right)}{\sum }𝕀\left(a_{k}=\text{request}\right) \end{array}$$ (4)

The count of completion events is indicated in Equation 5.


$$\begin{array}{l} C_{i}\left(t\right)=\underset{e_{k}\in W_{i}\left(t\right)}{\sum }𝕀\left(a_{k}=\text{completion}\right) \end{array}$$ (5)

where *I* denotes the indicator function, equal to 1 if the condition holds and 0 otherwise, *E*_*i*_(*t*) denotes the number of entry events, *R*_*i*_(*t*) denotes the number of request events, *C*_*i*_(*t*) is the number of completion events.

### 3.2 Engagement and completion rates

To normalize interaction intensity, engagement rate ρ_*i*_(*t*), is defined in Equation 6.


$$\begin{array}{l} \rho_{i}\left(t\right)=\frac{R_{i}\left(t\right)}{E_{i}\left(t\right)+\varepsilon } \end{array}$$ (6)

The completion rate κ_*i*_(*t*) is given in Equation 7.


$$\begin{array}{l} \kappa_{i}\left(t\right)=\frac{C_{i}\left(t\right)}{R_{i}\left(t\right)+\varepsilon } \end{array}$$ (7)

where ε > 0 ensures numerical stability.

### 3.3 Feature vector construction and normalization

The feature vector concatenation *V*_*f*_(*t*) that captures its behavior and characteristics in time *t* is represented in Equation 8.


$$\begin{array}{l} V_{f}\left(t\right)=\left[E_{i}\left(t\right),R_{i}\left(t\right),C_{i}\left(t\right),\rho_{i}\left(t\right),\kappa_{i}\left(t\right),s_{i}\right] \end{array}$$ (8)

Where *s*_*i*_ denotes static attributes of the resource *i* (e.g., location, type, capacity, or configuration parameters).

To ensure consistency, raw feature values are represented as *x*_*i,j*_(*t*), where *i* denotes the resource index, *j* denotes the feature dimension, and *t* denotes time. The corresponding normalized feature values are represented as ${\tilde{x}}_{i,j}\left(t\right)$. The vector forms are denoted by *x*_*i*_(*t*) and ${\tilde{x}}_{i}\left(t\right)$ for raw and normalized feature vectors, respectively. Normalized feature values are expressed in Equation 9.


$$\begin{array}{l} {\tilde{x}}_{i,j}\left(t\right)=\frac{x_{i,j}\left(t\right)-\mu_{j}\left(t\right)}{\sigma_{j}\left(t\right)+\epsilon } \end{array}$$ (9)

where μ_*j*_(*t*) denotes the mean value of the *j*-th feature computed across all resources at time *t*, σ_*j*_(*t*) denotes the standard deviation, and ϵ denotes the numerical stability constant and *x*_*i,j*_(*t*) denotes the original (raw) value of the *j*-th feature for resource *i* at time *t*.

For each normalized metric, *B*_*i*_(*t* − 1) denotes the adaptive baseline value at time (*t* − 1). It is computed using an exponentially weighted moving average (EWMA) in Equation 10.


$$\begin{array}{l} B_{i}\left(t\right)=\lambda x_{i}\left(t\right)\;+\left(1-\lambda \right)B_{i}\left(t-1\right) \end{array}$$ (10)

where λ ∈ (0, 1) controls the responsiveness of the EWMA to recent changes, λ*x*_*i*_(*t*) represents the weighted contribution of the current normalized metric, and (1−λ)*B*_*i*_ represents the contribution of the previous baseline value.

### 3.4 Trend score and detection rule

The standardized deviation or z-score relative to baseline *t*_*i*_(*t*) is quantified in Equation 11.


$$\begin{array}{l} t_{i}\left(t\right)=\frac{x_{i}\left(t\right)-B_{i}\left(t\right)}{\sigma_{i}\left(t\right)+\epsilon } \end{array}$$ (11)

Where σ_*i*_(*t*) denotes the standard deviation of the normalized metric, ϵ denotes a small positive constant to ensure numerical stability and avoid division by zero.

Threshold-based classification rule is formulated as a hypothesis test, which is given in Equation 12.


$$\begin{array}{l} \{\begin{array}{cc} t_{i}(t)\geq \theta^{+} & \text{Upward trend}\\ t_{i}(t)\leq -\theta^{-} & \text{Downward trend}\\ \text{otherwise} & \text{Normal behavior} \end{array} \end{array}$$ (12)

where θ^+^ denotes a positive threshold for detecting significant upward deviation, and θ^−^ denotes negative threshold for detecting significant downward deviation. Using enriched features, short-term demand forecasting ${\hat{D}}_{i}\left(t+h\right)$ is defined in Equation 13.


$$\begin{array}{l} {\hat{D}}_{i}\left(t+h\right)=f_{\theta }\left(D_{i}\left(t-p:t\right),\;{\tilde{\text{x}}}_{i}\left(t-p:t\right),\;t_{i}\left(t\right)\right) \end{array}$$ (13)

where *f*_θ_ denotes a parametric forecasting model, *D*_*i*_(*t* − *p* : *t*) denotes the historical demand sequence for the resource *i* from time *t* − *p* to *t*, *p* is a look-back window, ${\tilde{x}}_{i}\left(t-p:t\right)$ is the sequence of normalized feature vectors for the resource *i* over the same time window, and *t*_*i*_(*t*) is a current trend score, capturing deviation from baseline (used to enhance forecasting accuracy).

Therefore, the loss function L(θ) is used to train the parametric forecasting model *f*_θ_ which is calculated using Equation 14.


$$\begin{array}{l} \begin{array}{c} L\left(\theta \right)=\frac{1}{N}\underset{i,t}{\sum }w_{i}|\frac{D_{i}\left(t\;+\;h\right)\;-\;{\hat{D}}_{i}\left(t\;+\;h\right)}{D_{i}\left(t\;+\;h\right)+\epsilon }| \end{array} \end{array}$$ (14)

Where *D*_*i*_(*t* + *h*) is the actual (ground-truth) demand value for the resource *i* at a future time *t* + *h*.

## 4 Proposed AI-driven real-time trend analytics system

This intelligent trend analysis system is designed to help the city services of large cities in Kazakhstan (for example, Almaty) in operational decision-making based on the current situation. The system collects heterogeneous data in real time, such as transport sensor readings, medical visits, user actions on digital government portals, etc., and enriches them with contextual information and then identifies changes in the dynamics of key indicators of the urban environment (such as traffic congestion, patient flow, or user activity of electronic services). The insights gained are integrated into short-term forecasting models, which allows you to plan resources and adjust the work of services (redistribute traffic flows, strengthen medical teams, scale digital services, etc.) in accordance with current trends.

The AISSC framework, inspired by Kazakhstan's smart city environments, does not confine its architectural design to a specific city or country. The framework operates on generic streaming events, standardized feature representations, and service-agnostic analytical modules that are independent of local infrastructure implementations. Consequently, while Kazakhstan provides the motivating deployment scenario, the proposed methodology is intended for heterogeneous smart city environments with comparable IoT-enabled transportation, healthcare, e-government, and urban sensing services. The experimental evaluation therefore employs publicly available benchmark datasets that represent representative urban sensing and transportation characteristics rather than city-specific operational data.

The proposed AISSC framework was evaluated using a prototype implementation operating in a controlled real-time streaming experimental environment rather than being deployed within a production smart city infrastructure. The evaluation employed publicly available benchmark datasets that were continuously streamed to emulate real-time urban service operation under identical experimental conditions. Therefore, the reported forecasting accuracy, trend detection latency, and decision-support performance reflect controlled experimental validation of the proposed architecture rather than measurements collected from a live city-wide operational deployment. Nevertheless, the modular distributed architecture was specifically designed to facilitate future integration with operational smart city platforms and large-scale urban data infrastructures.

To demonstrate the practical operational efficacy of the proposed system, we present specific instances of intervention scenarios. In a transportation context, when the system detects a rapid increase in traffic congestion (positive trend score), the decision module may recommend adaptive traffic signal adjustments or temporary rerouting strategies. In a hospital setting, a swift increase in patient admission may need the dynamic reallocation of workers or the deployment of additional service units. In digital government services, a surge in user demands may lead to the automated expansion of server capacity or the prioritization of critical services. These examples demonstrate that the AISSC paradigm provides analytical insights and enables effective, context-sensitive interventions in the operations of smart cities.

**Figure 1:**
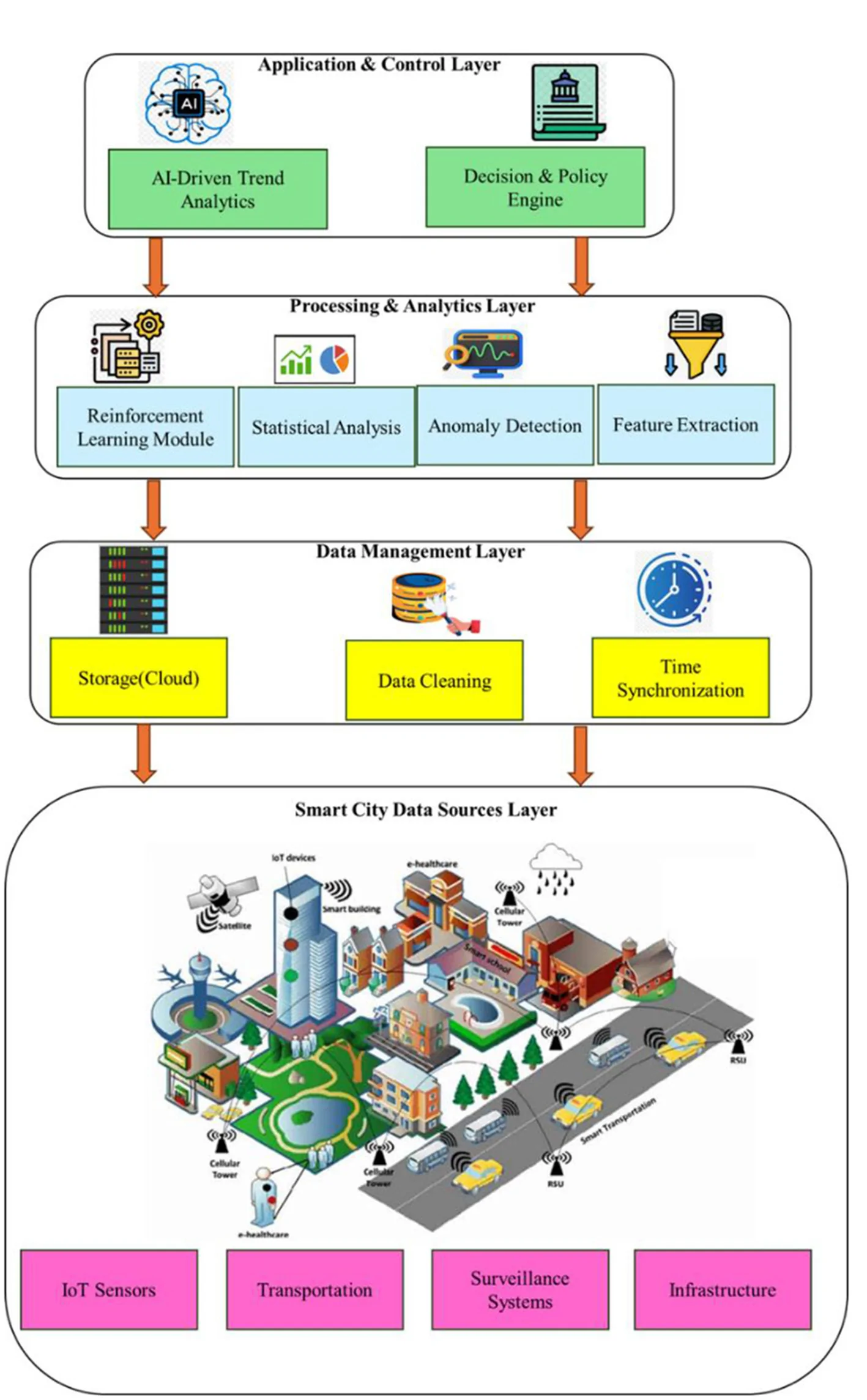
Smart city data analytics architecture and processing pipeline.

The framework incorporates several components to support large-scale and heterogeneous urban environments. These components include distributed data gathering, strong data management, powerful analytics, and intelligent application control. Because architecture is developed in a modular and hierarchical form, it is able to provide scalability, interoperability, and real-time decision-making across a wide variety of smart city domains. These domains include transportation, surveillance, infrastructure, and public services. Figure 1 depicts a comprehensive and layered architecture of a smart city analytics and decision-support system driven by artificial intelligence. The system that has been suggested is organized into five levels, each of which is logically independent from the other layers but is tightly related to the other layers.

### 4.1 Application and control layer

The application and control layer constitutes the pinnacle of abstraction within the architectural framework, facilitating the conversion of analytical results into practical intelligence. This layer encompasses two fundamental elements: the AI-powered trend analytics module and the decision and policy engine. The trend analytics module persistently scrutinizes processed data streams to discern enduring patterns, nascent trends, and behavioral alterations within urban systems, including traffic congestion, energy consumption irregularities, and public safety vulnerabilities. Subsequently, the decision and policy engine formulates adaptive strategies, policy suggestions, and operational controls, all of which are congruent with established objectives. It supports both automated and human-in-the-loop decision-making by enabling city managers to review, validate, and adjust system-generated actions. Consequently, this layer serves as a critical interface between data-driven analytics and governance, facilitating informed and responsive decision-making in real-world smart city environments.

### 4.2 Processing and analytics layer

The processing and analytics layer serves as the core intelligence of architecture, responsible for deriving actionable insights from the data that has been preprocessed. It utilizes sophisticated computational and analytical methodologies to convert raw inputs into valuable insights. This layer incorporates several critical functions, such as feature extraction, statistical analysis, anomaly detection, and a reinforcement learning module. These components collectively facilitate the identification of intricate patterns, the detection of deviations from anticipated behavior, and adaptive learning within dynamic environments, thus improving the system's analytical capacity and decision-support efficacy. Feature extraction serves to convert unprocessed sensor and system data into concise, informative representations, thereby facilitating their application in machine learning models. Statistical analysis furnishes both descriptive and predictive perspectives by uncovering correlations, distributions, and temporal patterns inherent in the data. Anomaly detection mechanisms are employed to monitor deviations from anticipated behavior, thereby enabling the early identification of faults, security threats, and irregular occurrences. Furthermore, a reinforcement learning module enhances the system's adaptability by continuously learning the best actions based on environmental feedback. Therefore, this ability allows the system to gradually improve its performance and responsiveness in changing urban environments.

In the present implementation, the reinforcement learning component is realized using a tabular Q-learning algorithm. Q-learning was selected because it is a model-free reinforcement learning method with low computational complexity, stable online learning behavior, and straightforward integration into streaming decision-support systems. The algorithm incrementally updates state-action value estimates using environmental feedback without requiring an explicit model of urban system dynamics, making it well suited to continuously evolving smart city environments. Furthermore, compared with more computationally intensive deep reinforcement learning approaches, such as Deep Q-Networks (DQN) or policy-gradient methods, Q-learning provides fast policy adaptation, low memory requirements, and improved interpretability, which are important for latency-sensitive operational decision support. Although more advanced deep reinforcement learning algorithms may provide additional benefits in large-scale continuous state spaces, their investigation is beyond the scope of the present work and will be considered in future research.

Although the proposed framework adopts Q-learning as the reinforcement learning component, alternative operational decision-making strategies were also considered during the system design stage. These include static rule-based policies, threshold-driven control mechanisms, greedy action selection, and supervised classification-based decision support. Rule-based and threshold-driven approaches provide low computational cost but cannot adapt automatically to continuously evolving streaming environments. Greedy strategies optimize only immediate operational objectives without considering long-term system performance, while supervised learning methods require labeled decision data and do not naturally support sequential interaction with dynamic environments. Because the primary objective of this work is to demonstrate the feasibility of integrating adaptive decision support into a unified real-time streaming analytics framework, Q-learning was selected as an appropriate baseline reinforcement learning method owing to its low computational complexity, online learning capability, and suitability for sequential decision optimization. A comprehensive quantitative comparison with alternative decision-making strategies constitutes an important direction for future investigation. Table 2 shows the qualitative comparison of alternative decision-making strategies considered for adaptive smart city operation.

**Table 2:** Qualitative analysis of multiple decision-making processes evaluated for adaptive smart city operations.

Strategy	Adaptivity	Sequential learning	Real-time suitability	Computational cost
Rule-based policy	Low	No	Medium	Very low
Threshold policy	Low	No	Medium	Very low
Supervised classifier	Medium	No	Medium	Medium
Greedy policy	Medium	Limited	High	Low
Q-learning (AISSC)	High	Yes	High	Low

The present work focuses on validating the effectiveness of the proposed integrated streaming analytics architecture rather than performing an exhaustive benchmark of reinforcement learning algorithms or operational policy optimization methods.

Within the proposed Q-learning framework, the reinforcement learning agent interacts with the smart city environment through well-defined state, action, and reward representations. The state is composed of the current normalized feature vector of each monitored resource, including engagement rate, completion rate, demand level, trend score, and contextual resource attributes extracted from the streaming analytics module. The action corresponds to selecting an adaptive operational recommendation, such as maintaining the current operational policy, increasing or decreasing resource allocation, adjusting traffic-control parameters, prioritizing critical digital services, or issuing an early monitoring alert depending on the application domain. The reward is designed to encourage timely and reliable operational decisions by maximizing service efficiency and trend prediction performance while minimizing false alarms, service congestion, response latency, and resource over-utilization. At each step, the environment generates a scalar reward that reflects the experienced operational outcome. The agent, using Q-learning, can iteratively improve its decision policy through ongoing interaction with the streaming environment.

Smart city environments are inherently non-stationary because user behavior, transportation demand, healthcare utilization, weather conditions, public events, and digital service usage continuously evolve. The proposed AISSC framework addresses this challenge through continuous online interaction between the reinforcement learning agent and the streaming analytics module. As newly arriving events modify the normalized feature vectors, adaptive EWMA baselines, trend scores, and contextual state representation, the reinforcement learning agent receives updated state observations and corresponding reward signals without interrupting system operation. Such repeated Q-learning updates gradually improve previously learned action-value estimates and allow the policy to adapt to changing behavior patterns over time, instead of relying on fixed offline models. Furthermore, by continuously updating the state representation with sliding-window analytics and adaptive feature normalization, stale behavioral information is naturally de-emphasized, enabling the decision policy to be sensitive to slow concept drift while robust to short-term fluctuations.

To prevent unstable or undesirable operational recommendations, the proposed AISSC framework incorporates multiple safety mechanisms within the decision-support pipeline. First, reinforcement learning actions are only triggered when the streaming analytics module generates statistically validated trend information based on adaptive EWMA baselines and hypothesis testing, reducing the effect of transient noise and random fluctuations. Secondly, the reward function is designed to penalize false alarms, unnecessary resource reallocation, response latency and resource over-utilization to discourage an unsafe operational behavior. Third, the policy recommendations are generated under pre-specified operational constraints defined by domain-specific service policies, avoiding infeasible control actions. Fourth, the application and control layer supports human-in-the-loop supervision, which enables city operators to review, validate, modify or reject automatically generated recommendations before deployment in safety-critical scenarios. Together, these safeguards enhance policy stability, operational reliability and practical applicability in real-world smart city settings.

The Q-learning value function is updated in Equation 15.


$$\begin{array}{l} Q\left(s_{t},a_{t}\right)\leftarrow Q\left(s_{t},a_{t}\right)+\alpha \left[r_{t}+\gamma \underset{a^{\prime }}{\max}Q\left(s_{t+1},a^{\prime }\right)-Q\left(s_{t},a_{t}\right)\right] \end{array}$$ (15)

where *Q*(*s*_*t*_, *a*_*t*_) denotes the estimated action-value function, α is the learning rate, γ is the discount factor, *r*_*t*_ is the immediate reward received after executing action *a*_*t*_ in state *s*_*t*_, and *s*_*t*+1_ represents the next observed state.

### 4.3 Data management layer

**Figure 2:**
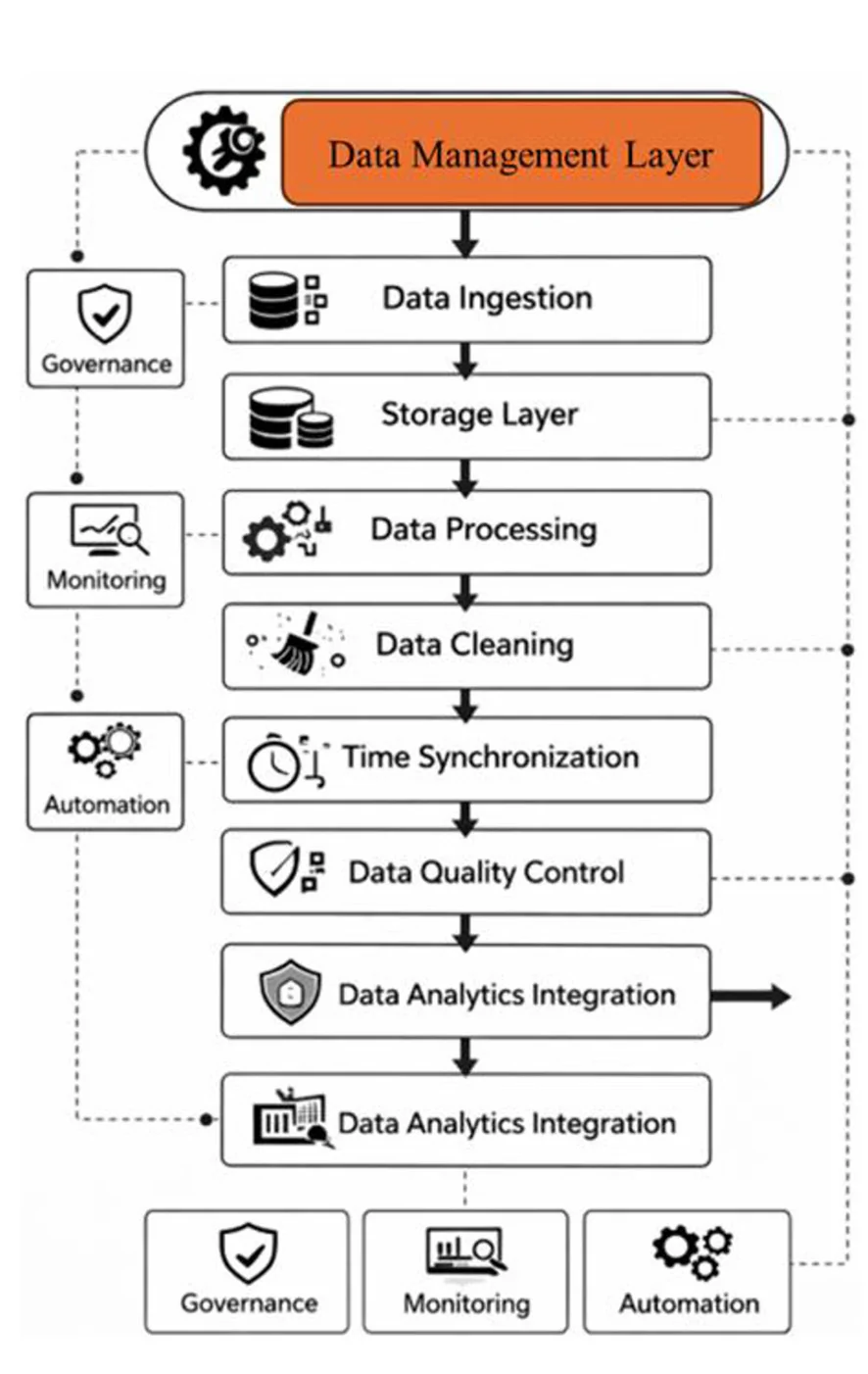
Data management pipeline illustrating sequential stages from data ingestion to analytics integration with integrated governance, monitoring, and automation mechanisms.

The data management pipeline, illustrated in Figure 2, encompasses sequential stages from data ingestion to analytics integration. To ensure superior analytical results, meticulously defined protocols for data cleansing, transformation, and validation are essential. This pipeline is designed for efficient data processing, emphasizing governance, supervision, and automation.

Consequently, the objective is to ensure reliability, scalability, and adherence to all pertinent regulations. Data acquisition involves gathering information from various sources, including Internet of Things (IoT) sensors, software applications, databases, and external systems. These sources can provide data that is structured, semi-structured, or unstructured. Initially, a validation phase filters out invalid or corrupted data. Efficient ingestion processes are then used to maximize throughput and minimize latency in continuous data streams. The collected data is stored using a scalable and secure infrastructure. This includes data warehouses, distributed databases, or cloud storage systems. This setup allows for both real-time and historical data processing (Kumar et al., 2022). After collecting data, it undergoes transformation, integration, and analysis to find important patterns, which then supports further investigation. Data cleaning protocols are essential for maintaining data integrity and reducing errors in analysis; these protocols involve removing duplicates, outliers, noise, and missing values.

The proposed data management layer adds additional mechanisms for dealing with incomplete and unreliable data streams to improve the robustness of streaming analytics in realistic operating scenarios. In the case of transient gaps, missing observations are handled through short-term forward propagation and for longer interruptions, statistically consistent imputation is achieved by means of adaptive EWMA estimates. Failures of sensors are detected through the continuous validation of the availability and consistency of data; persistent failures lead to the exclusion of unreliable streams until valid observations are resumed. Timestamp-based buffering and temporal synchronization reduce the effect of communication delays by enabling slightly delayed events to be correctly aligned prior to feature extraction. The normalization of streaming measurements is preceded by edge-level filtering and statistical validation to reduce noisy streaming measurements before normalization, preventing abnormal sensor readings from propagating into downstream trend detection, forecasting, and reinforcement learning modules. Moreover, using distributed computing technologies helps improve both scalability and efficiency when handling large datasets. In complex data environments, it's crucial to synchronize time. This ensures that events are aligned correctly, which is necessary for reliable correlation analysis. After synchronization, validation is essential for maintaining data consistency, completeness, and accuracy. As a result, this process improves the quality of the analyses that follow. The solid data is the bedrock of strong analytics, machine learning, and visualization. This, in turn, enables the identification of trends, accurate forecasting, and well-informed decisions, all facilitated through dashboards and reporting tools. The architecture's design integrates extensive automation, monitoring, and governance protocols throughout the entire data pipeline. Data governance protocols are implemented to guarantee secure data access, safeguard privacy, and ensure adherence to relevant regulatory frameworks. Continuous monitoring allows for the prompt identification of potential bottlenecks and system failures, thereby enabling swift corrective actions. Automation minimizes the need for manual intervention, thereby improving operational efficiency, scalability, and overall system reliability. Consequently, these interconnected mechanisms collectively empower the pipeline to effectively manage substantial data streams within fluctuating operational environments. The scalability of data management is fundamentally tied to the complexity of its system architecture. This framework is designed to optimize both data acquisition and processing, enabling advanced analytics while upholding security protocols, operational efficiency, and data integrity. Furthermore, structured processing phases and integrated control mechanisms refine analytical pipelines, enabling real-time decision-making and augmenting the system's overall intelligence.

Furthermore, the architecture facilitates the advancement of large-scale AI-driven data ecosystems, smart city infrastructures, and IoT platforms, as illustrated in Figure 2.

### 4.4 Smart city data sources layer

The smart city data sources layer constitutes the foundational tier of the proposed AISSC architecture, enabling the continuous acquisition and integration of varied, high-frequency urban data streams. This layer aggregates data from a wide spectrum of sources, including Internet of Things (IoT) devices, intelligent transportation systems, surveillance infrastructures, digital government platforms, and healthcare service networks. It supports a range of communication technologies. This includes 5G and 4G cellular networks, as well as established wireless protocols like IEEE 802.11 and IEEE 802.22. The result is reliable, large-scale connectivity across sprawling urban environments. The combination of data from different sources and types provides a detailed and thorough view of how cities work. This is crucial for real-time analysis and making decisions that can change based on new information.

To handle the challenges of large amounts of data that come in quickly, are very big, and are varied, this layer uses a mix of edge and cloud computing. Edge nodes, strategically positioned near data sources, handle operations that are sensitive to latency. These operations include event filtering, noise reduction, short-window aggregation, and preliminary feature computation. This localized processing approach significantly reduces communication demands, allowing for quick responses to events that require immediate action. Cloud-based infrastructure, at the same time, provides scalable storage, distributed processing power, and advanced analytical capabilities. These capabilities include model training, historical data analysis, and the ability to combine data from different domains. This dual-layered processing strategy ensures an optimal blend of speed and responsiveness. This is crucial for streaming analytics in the constantly changing environments of smart cities.

The proposed AISSC framework is designed to maintain continuous operation in case of temporary disruptions in the network connectivity between the edge and cloud layers. When there is a communication outage, edge nodes keep performing latency-sensitive operations such as event filtering, sliding-window aggregation, feature extraction, and preliminary trend monitoring using the resources available locally. For incoming events, the events are buffered temporarily along with timestamp metadata and synchronized on reestablishment of connectivity. This way, the cloud layer can reconstruct the entire event sequence without loss of temporal consistency. Adaptive EWMA baselines and edge sliding-window analytics allow for continuous operation of short-term monitoring and local decision support, even when intermittent communication failures occur. When connectivity is restored, buffered events are securely uploaded to the cloud for historical storage, global model updates and cross-domain analytics. Thus, short network outages affect mainly the centralized analytical updates, but not the local monitoring and the operational continuity. Moreover, architectural design supports reliable data transmission, interoperability, and the ability to recover from errors.

This is achieved through standardized communication interfaces, adaptive routing protocols, and protocol abstraction layers. These characteristics facilitate the seamless integration of diverse data sources, concurrently upholding consistency, reliability, and scalability across fluctuating network environments. Furthermore, the incorporation of multi-modal sensing—encompassing spatial, temporal, and contextual data—augments situational awareness and supports the identification of intricate urban patterns that manifest over time and space. Within the AISSC framework, the Smart City Data Sources Layer functions as the initial stage of a unified, end-to-end data processing pipeline, thereby enabling structured workflows that encompass data ingestion, validation, cleaning, temporal alignment, and feature extraction. In particular, temporal synchronization mechanisms ensure consistent alignment of asynchronous data streams originating from diverse sources, which is essential for accurate correlation analysis and trend detection. Data preprocessing steps, which mitigate noise, rectify missing values, and resolve inconsistencies, are fundamental to ensuring data quality and reliability. These structured and normalized feature representations subsequently serve as the foundation for analytical modules, encompassing statistically informed trend detection, short-term demand forecasting, and decision support systems predicated on reinforcement learning. From a systems design perspective, layered and modular architecture significantly improves scalability, maintainability, and extensibility. This distributed approach to gathering and preparing data supports horizontal scaling. As a result, it can handle increasing data volumes and the addition of new urban services. Moreover, the way data sources and communication methods are abstracted allows for the easy integration of new technologies and future data streams, which means that major changes to the system's structure aren't needed. Adaptability is particularly important in the practical application of smart cities, considering the ever-changing requirements of infrastructure and services.

The proposed AISSC framework is based on a distributed and layered processing architecture that is intended to support very large volume of concurrent events generated by heterogeneous urban services. Event streams received from transportation systems, healthcare services, e-government platforms, IoT sensors, and surveillance infrastructures are separately managed by applying resource-specific sliding windows before they are integrated into the common analytical pipeline. This partitioned processing approach enables parallel feature extraction, adaptive trend detection and forecasting on multiple services with reduced inter-service processing dependencies. Additionally, edge-level preprocessing decreases communication overhead by filtering, aggregating, and validating streaming events before cloud-level analytics, thereby avoiding bottlenecks from centralized processing. Though this study does not empirically evaluate workloads of millions of concurrent events, the proposed modular architecture is intended to enable horizontal scaling through additional processing nodes and distributed storage resources as event volume and diversity of services grows.

This layer supports the integration of contextual and external data sources, such as weather information, public events, social media activity, and policy-related data. The inclusion of these additional data sources enhances the feature space, which in turn improves the predictive and analytical capabilities of the overall system. This capability aligns with the AISSC's objective of acquiring both operational data and contextual factors influencing urban conduct and service requirements. Crucially, the Smart City Data Sources Layer is designed with data governance, security, and privacy as core design tenets. Access control, data encryption, and adherence to regulatory standards are integral to the data acquisition and transmission phases. These protocols safeguard sensitive urban data while ensuring that authorized analytical processes retain access, thus fostering reliable and accountable data-driven decision-making.

The AISSC framework allows for real-time analytics by processing streaming events with anonymized resource-level representations instead of personally identifiable information, thereby protecting the privacy of citizens. Downstream analytical modules do not persist or use user identities but rather convert event streams into aggregated engagement, completion, and demand metrics in sliding time windows before feature extraction. Raw operational data is not available according to role-based access control. Encrypted data transmission and secure storage ensure the security of data acquisition and processing. As a result, the suggested framework conducts trend detection, forecasting, and reinforcement learning-based decision support via aggregated statistical representations, minimizing exposure of sensitive citizen-generated and service-usage information while remaining compliant with applicable data governance and privacy requirements.

AISSC's modular and service-agnostic architecture is inherently portable to smart cities with varying infrastructure capabilities, governance arrangements and data ecosystems. Although the proposed AISSC framework is motivated by Kazakhstan's smart city applications, its modular and service-agnostic architecture is designed for deployment in heterogeneous urban environments beyond a single geographical region. The framework abstracts data acquisition through standardized streaming representations and analytical interfaces, enabling adaptation to smart cities with different infrastructure capabilities, governance arrangements, and data ecosystems. The smart city data sources layer abstracts data acquisition from specific technologies enabling the ingestion of heterogeneous inputs from transportation systems, healthcare services, e-government platforms and IoT infrastructures. Standardized feature representations, temporal synchronization mechanisms and adaptive normalization techniques further enable the framework to operate in a variety of data distribution and service configuration contexts. The layered architecture allows for the independent deployment or extension of individual modules, according to local technological capabilities and governance arrangements. This allows AISSC to be adapted to cities with different levels of digital maturity without major changes to the overall analytical workflow.

The AISSC architecture he proposed is intentionally designed to support the continuous evolution of smart city ecosystems through its modular, service-agnostic architecture. New urban services, such as smart parking, environmental monitoring, energy management, waste collection, emergency response, or future IoT applications, can be incorporated by implementing lightweight data adapters that transform incoming events into the standardized streaming event representation used throughout the framework. Since downstream modules—including temporal synchronization, feature extraction, trend detection, forecasting, and reinforcement-learning-based decision support—operate on normalized feature representations rather than application-specific formats, they require no architectural redesign when additional services are introduced. This abstraction allows incremental expansion of systems while keeping interoperability, scalability and analytical consistency across heterogeneous urban infrastructures. Consequently, this foundational layer provides a strong and adaptable basis for real-time, AI-enhanced urban intelligence. Facilitating a continuous, high-quality data stream into the analytical core of the AISSC framework, it underpins proactive urban management, adaptive policy development, and efficient resource distribution. The system's ability to adapt to changing urban environments is significantly improved by using data collection, real-time analysis, and intelligent decision-making. The Smart City Data Sources Layer is a crucial element for the development of future smart cities. These cities are distinguished by their ability to quickly analyze data, use data to improve governance, and offer services that are both resilient and adaptable.

### 4.5 Real-time data ingestion and feature engineering

**Figure 3:**
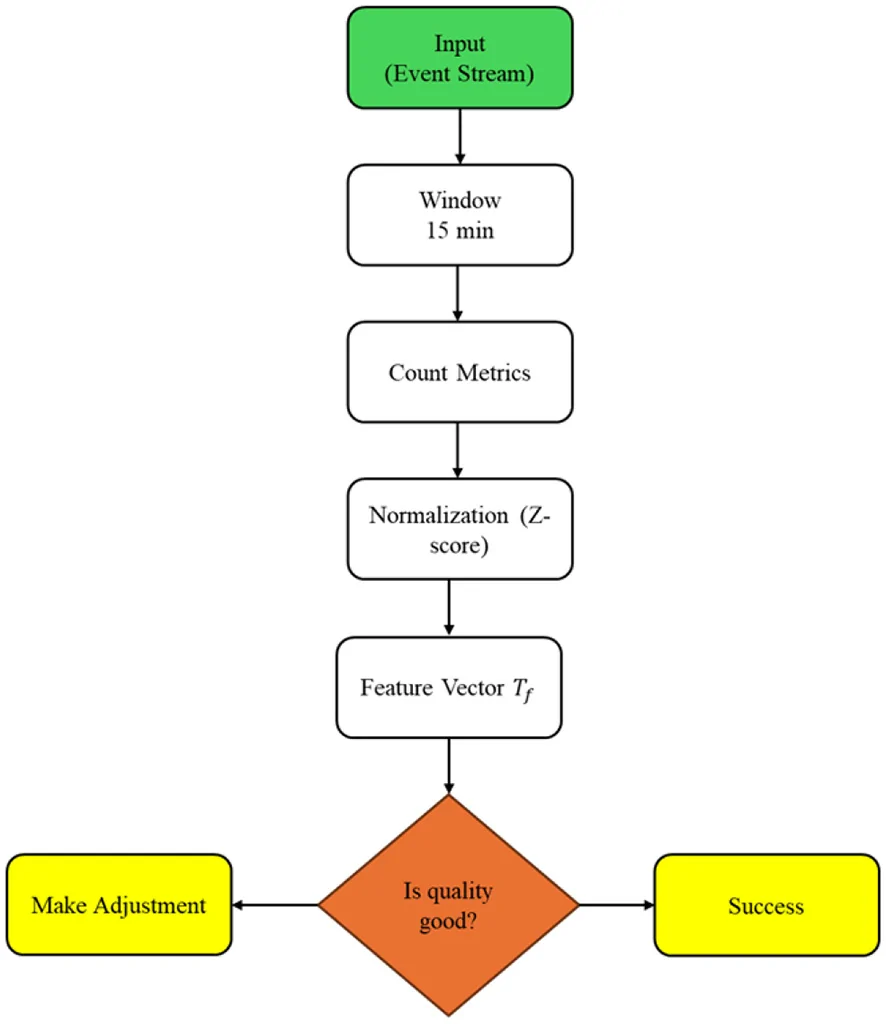
Real-time data ingestion and feature engineering pipeline for Smart City services.

In this module, streaming data from city sensors and service logs are continuously collected and transformed into structured features at multiple levels, including transportation routes, e-government services, and healthcare facilities depicted in Figure 3. Each incoming event is represented as *e*_*k*_ = (*u*_*k*_, *i*_*k*_, *a*_*k*_, *c*_*k*_, *t*_*k*_).

For each resource *i* at time *t*, a Sliding event window (Δ) is maintained. Thus, the set of all events *E*(*i*, Δ)_*t*_ associated with the resource *i* can be determined in Equation 16.


$$\begin{array}{l} E{\left(i,\Delta \right)}_{t}=\left\{e_{s}:e_{v}\in \left[et-\Delta ,et\right]\right\} \end{array}$$ (16)

where, *e*_*s*_ denotes the single event.

The variable *t* represents the current time, Δ defines the window duration (e.g., 15 minutes), and *t*_*e*_ denotes the timestamp of an individual event *e*. Only events satisfying the condition *e*_*v*_ ≥ *t* − Δ are included in the sliding window, allowing the model to capture short-term dynamics of urban activity. In the upper part of this window, the main indicators are the count of entry events *N*_*entry*_(*i*)_Δ_ associated with the resource *i* within the current sliding window Δ calculated in Equation 17.


$$\begin{array}{l} N_{\text{entry}}{\left(i\right)}_{\Delta }=\underset{e_{s}\in E{\left(i,\Delta \right)}_{t}}{\sum }1\left(a_{e}\right) \end{array}$$ (17)

The summation is defined over *e*_*s*_ ∈ *E*(*i*, Δ)_*t*_, where 1(*a*_*e*_) = 1 if *a*_*e*_ is an entry action, and 1(*a*_*e*_) = 0 otherwise. The resulting value corresponds to the count of request events for the resource *i* over the time window Δ. Thus, the number of active requests for the source can be calculated using Equation 18.


$$\begin{array}{l} N_{\text{request}}{\left(i\right)}_{\Delta }=\underset{e_{s}\in E{\left(i,\Delta \right)}_{t}}{\sum }1\left(a_{e}\right) \end{array}$$ (18)

where *N*_request_(*i*)_Δ_ is the number of active requests or usage events for the resource *i* for the sliding window Δ, *E* denotes a set of events.

The summation runs over all events in the window, and 1(*a*_*e*_) returns 1 if the event *e* is an active service request or an intermediate engagement (such as a submitted form or a vehicle moving through an intersection), and 0 otherwise. This metric reflects a more advanced level of engagement than entry events. Thus, the count of completion events *N*_complete_(*i*)_Δ_ is defined in Equation 19.


$$\begin{array}{l} N_{\text{complete}}{\left(i\right)}_{\Delta }=\underset{e_{s}\in E{\left(i,\Delta \right)}_{t}}{\sum }1\left(a_{e}\right) \end{array}$$ (19)

The summation runs over all events in the window, and **1**(*a*_*e*_) equals 1 whenever the single event *e*_*s*_ corresponds to a completed service or transaction for *i* (such as a successfully processed request or a completed treatment), and 0 otherwise. This quantity directly reflects the realized demand for the resource *i* over the chosen short-term horizon. Using these counts, the engagement rate *ER*_*i*_(*t*) is provided in Equation 20.


$$\begin{array}{l} ER_{i}\left(t\right)=\frac{N_{\text{request}}{\left(i\right)}_{\Delta }}{N_{\text{entry}}{\left(i\right)}_{\Delta }+\epsilon } \end{array}$$ (20)

A small constant ϵ is added for numerical stability to avoid division by zero when the number of entries is extremely low. Intuitively, *ER*_*i*_(*t*) measures the fraction of initial interactions that lead to active usage requests, reflecting how engaging or accessible the service is to users. Therefore, completion rate is calculated in Equation 21.


$$\begin{array}{l} CR_{i}\left(t\right)=\frac{N_{\text{complete}}{\left(i\right)}_{\Delta }}{N_{\text{request}}{\left(i\right)}_{\Delta }+\epsilon } \end{array}$$ (21)

The completion rate quantifies the probability that an initiated request or engagement with a resource *i*eventually results in a successful completion, thus linking user interest to realized service delivery.

A feature vector concatenation *V*_*f*_*i*__(*t*) is formed for each resource in *t*, combining indicators calculated based on event data and its static characteristics given in Equation 22.


$$\begin{array}{l} V_{f_{i}}\left(t\right)\;=\left[\begin{array}{c} N_{\text{entry}}{\left(i\right)}_{\Delta },N_{\text{request}}{\left(i\right)}_{\Delta },N_{\text{complete}}{\left(i\right)}_{\Delta },\\ ER_{i}\left(t\right),\\ CR_{i}\left(t\right),s_{i} \end{array}\right] \end{array}$$ (22)

Where *ER*_*i*_ denotes the engagement rate capturing exposure efficiency (how many initial entries convert into active usage); *CR*_*i*_ is the completion rate capturing fulfillment probability, and *s*_*i*_ denotes the static attributes of the resource *i* (such as type of service—transport, eGov, health—as well as capacity or size, and location or region). This feature vector serves as the unified numerical representation of recent user behavior and resource characteristics, and it forms the input to downstream analytical modules.

To alleviate feature redundancy and meanwhile maintain informative representations, the feature engineering of the proposed framework combines normalization, stability analysis and informativeness evaluation. Algorithm 1 finds features with very low temporal variability or with insufficient information content using stability-based and entropy-based quality assessment. Metrics derived from such as engagement and completion rates also capture the relationships between raw event counts, thus reducing the need to propagate multiple highly correlated variables to downstream analytical modules. The resulting feature representation is therefore designed to retain complementary behavioral information while limiting redundant or low-informative attributes that could adversely affect trend detection and forecasting performance.

Algorithm 1 Real-time data ingestion and feature engineering for trend analytics. 
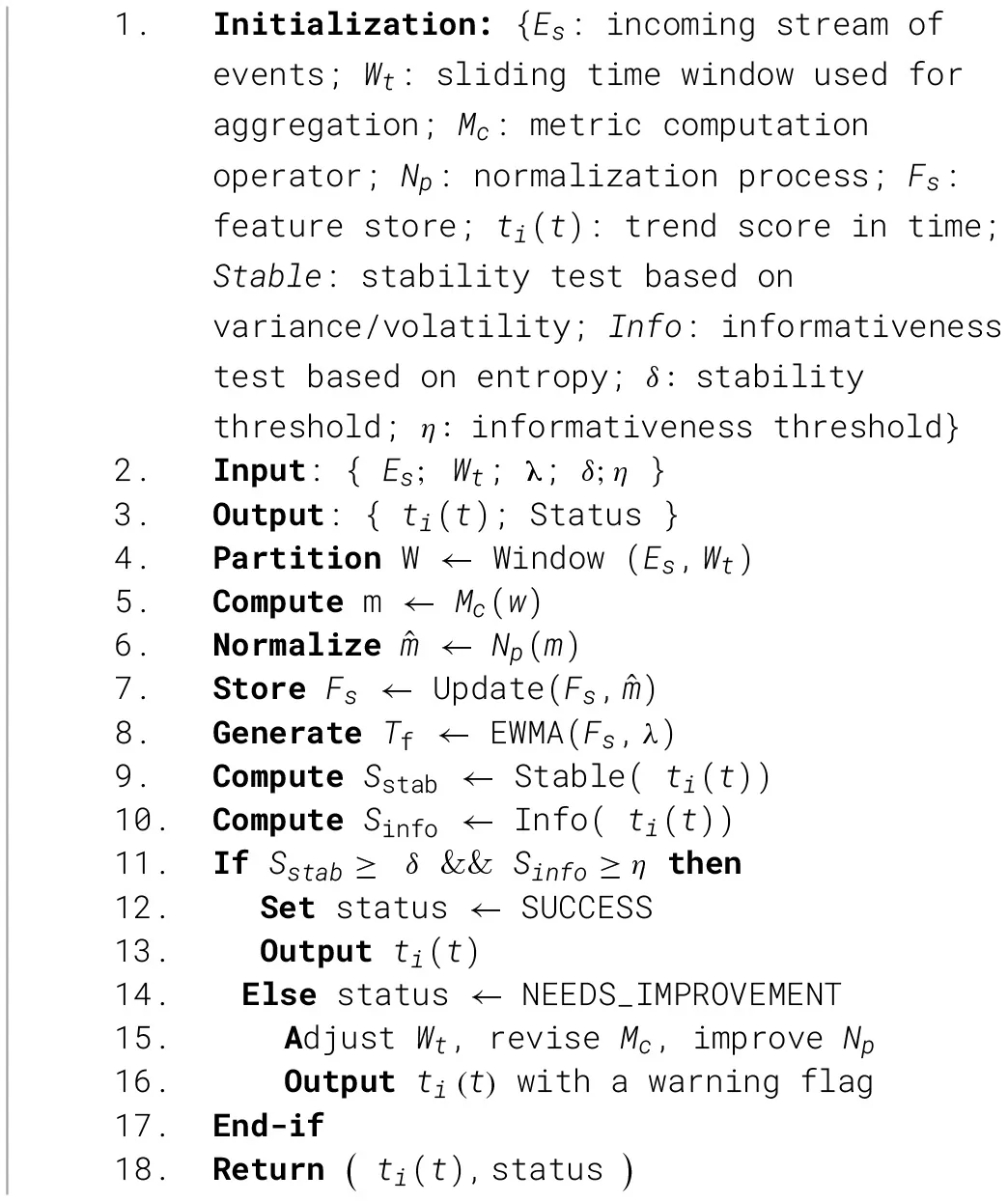

The temporal granularity metric *G*_Δ_(*f*) for the feature *f* is quantified by measuring the variance of features over time on a short window scale. Therefore, temporal granularity metric can be determined using Equation 23.


$$\begin{array}{l} G_{\Delta }\left(f\right)=E_{t_{0}}\left[Var_{t\in \left[t_{0},t_{0}+\Delta \right]}\left(x_{i}\left(t\right)\right)\right] \end{array}$$ (23)

*Var*_*t*∈[*t*_0_, *t*_0_+Δ]_(*x*_*i*_(*t*)) is the variance of the feature for resource *i* computed over a short time interval (for example, over several minutes), starting at the time *t*_*o*_. The expectation 𝔼_*t*_0__ is taken over different starting positions *t*_0_ for such windows in time. This metric *G*_Δ_(*f*) measures how much a feature fluctuates within short time intervals. Higher values correspond to higher temporal variability, while lower values correspond to more stable usage.

To quantify the magnitude of temporal fluctuations of streaming behavioral signals rather than their information content, the temporal granularity metric was specifically designed with short-window variance as the core value. Variance is a computationally cheap measure that is well suited to high frequency streaming environments. It allows for fast updates in sliding windows and is highly sensitive to sudden changes in service usage. Entropy based or information theoretic measures instead are mainly measures of uncertainty or complexity of distributions. These measures usually require reliable estimation of the underlying probability distributions over sufficiently large windows of observations. Such requirements introduce additional computational overhead and may reduce responsiveness under strict real-time latency constraints. Therefore, variance was selected as an appropriate trade-off between computational efficiency, temporal sensitivity, and analytical robustness for continuous smart-city streaming analytics, whereas entropy-based measures are considered an interesting direction for future extensions of the framework.

The comparability of features *f*_*c*_ across resources is also quantified by examining the average pairwise distance between normalized feature vectors which is given in Equation 24.


$$\begin{array}{l} f_{c}=E_{i\neq j}\left[∥{\tilde{x}}_{i}\left(t\right)-{\tilde{x}}_{j}\left(t\right){∥}_{2}\right] \end{array}$$ (24)

where ${\tilde{x}}_{i}\left(t\right)$ and ${\tilde{x}}_{j}\left(t\right)$ are the normalized feature vectors for resources *i* and *j* at time *t*; ∥·∥_2_ denotes the Euclidean norm (L2 norm) measuring distance in feature space, and the expectation is taken over all pairs of distinct resources *i* ≠ *j*. The metric characterizes the degree of similarity between normalized resource representations. Lower values correspond to similar usage patterns, whereas higher values indicate differences in resource behavior.

Algorithm 1 illustrates real-time data input and feature engineering for trend analysis.

The workflow of trend detection and service monitoring is shown in Algorithm 2. In Step 1 the variables and parameters that will be used throughout the algorithm are initialized. In Step 2 the input feature vectors and system parameters are defined and in Step 3 the expected outputs are specified. In Step 4 the adaptive baseline behavior is calculated based on the current normalized feature vectors. In Step 5 the deviation of the observed features from the baseline is estimated to quantify the volatility and behavioral drift. In Step 6 trend scores and anomaly indicators are created based on these deviations. In Step 7 the trend outputs are converted into aggregated service level performance metrics.

Algorithm 2 Trend detection and service monitoring. 
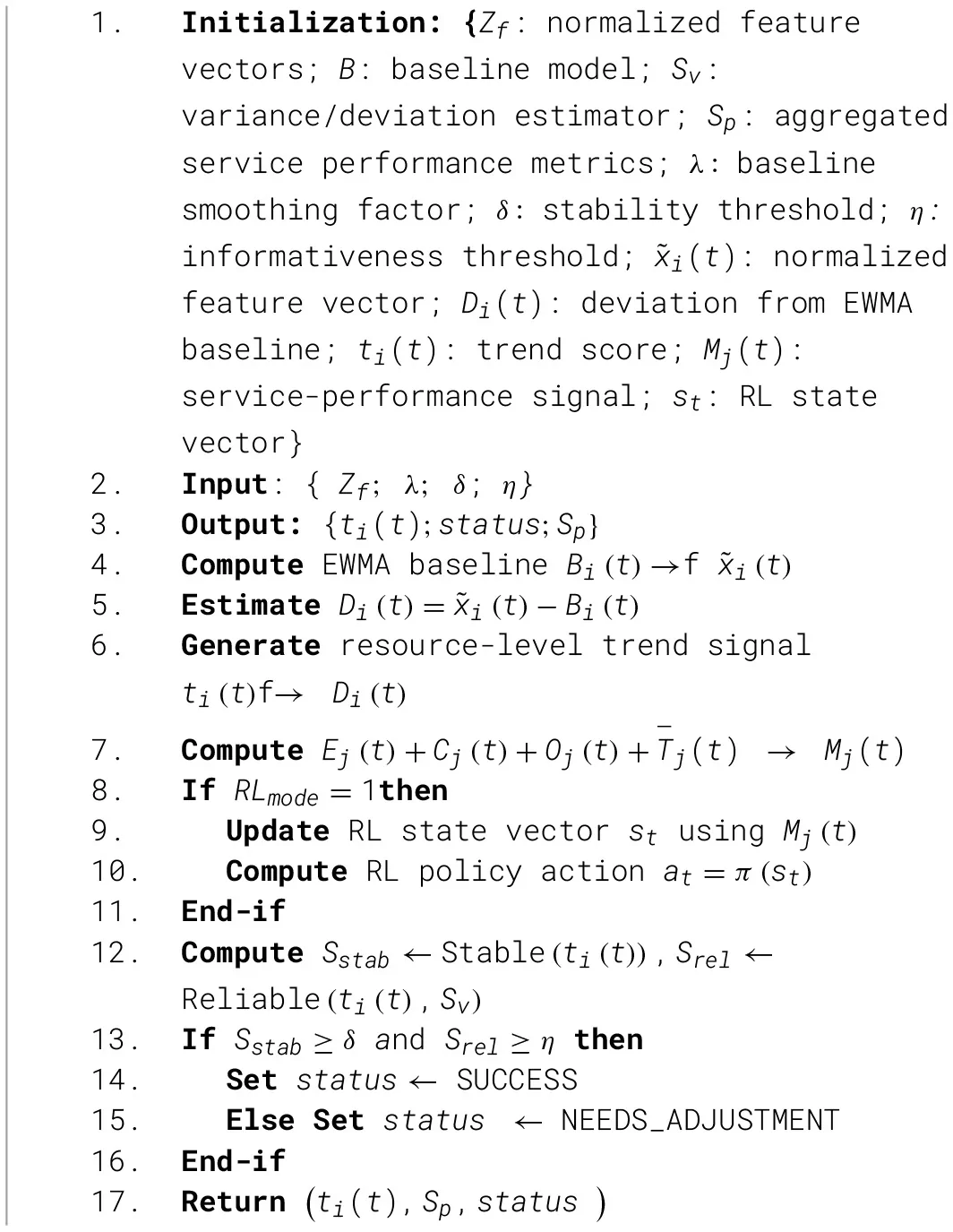

In Step 8, the algorithm checks whether the reinforcement learning module is enabled. If reinforcement learning is enabled, Step 9 updates the current reinforcement learning state with the latest service metrics, enabling the agent to observe the operational environment. Step 10 calculates the corresponding adaptive policy action or monitoring recommendation. Step 11 indicates the end of the optional reinforcement learning stage.

In Step 12, the quality and reliability of the generated trend signals are evaluated. Step 13 checks if the detected trends satisfy the pre-specified stability and reliability thresholds. In the case of satisfying conditions, Step 14 assigns SUCCESS as the monitoring status, otherwise Step 15 assigns NEEDS_IMPROVEMENT as the status, which indicates that the trends are not sufficiently reliable or the detected signals are not stable. Steps 16 and 17 close the conditional decision process. Step 18 presents the generated monitoring output along with the corresponding status indicators.

In summary, Algorithm 2 provides a systematic and reproducible framework for real-time trend detection, adaptive monitoring, and reinforcement learning-assisted service management in a smart city context.

The hypotheses, lemmas, and corollaries are not introduced as abstract theoretical extensions, but rather as lightweight analytical justifications intended to formalize the behavior of the proposed streaming analytics framework under practical smart city conditions. Their purpose is to clarify the statistical properties of temporal aggregation, feature normalization, trend detection stability, and reinforcement-learning-driven adaptation within the AISSC pipeline. These formal statements are included primarily to improve reproducibility, interpretability, and analytical consistency between the streaming architecture and the observed empirical behavior

Hypothesis-1: Short-window streaming aggregation with window length Δ preserves higher temporal granularity of usage signals than coarse-grained daily aggregation.

Proof: Let *F*_daily_(*d*) denote a daily aggregated feature for the day *d*, and let *F*_Δ_(*d*) be the same feature computed over windows of size Δ≪1 day. Under non-stationary usage patterns with intraday peaks (e.g., rush-hour traffic surges or lunchtime service spikes), the variance of the signal at the short time satisfies which is given in Equation 25.


$$\begin{array}{l} \text{Var}\left(F_{\Delta }\left(d\right)\right)>\text{Var}\left(F_{\text{daily}}\left(d\right)\right) \end{array}$$ (25)

Here Var[*F*_Δ_(*d*)] denotes the variability of the feature when aggregated over short windows, and Var[*F*_daily_(*d*)] is the variability of the same feature at daily resolution. This inequality compares the temporal variability retained at different aggregation resolutions. Thus, the expected temporal granularity metric *G*_Δ_(*f*) is higher than its daily-aggregated counterpart *G*_daily_(*f*). In other words, short-window aggregation captures finer temporal patterns in usage dynamics.

Hypothesis-2: Z-score normalization applied at each time step improves cross-resource comparability by bounding the scale of each feature dimension and stabilizing pairwise distances.

Proof: Without normalization, the pairwise distance between resources can be dominated by high-variance features. After applying the normalization, every feature satisfies which is given in Equation 26.


$$\begin{array}{l} E_{i}\left[{\tilde{x}}_{i}\left(t\right)\right]=0,\;{\text{Var}}_{i}\left[{\tilde{x}}_{i}\left(t\right)\right]=1 \end{array}$$ (26)

where E_*i*_ denotes expectation over the set of resources, an Var_*i*_ denotes variance across resources Var_*i*_. This expresses that after normalization; each feature dimension is centered (zero mean) and scaled to unit variance.

Therefore, the expected squared distance between any two resources becomes is given in Equation 27.


$$\begin{array}{l} E_{i\neq j}\left[∥{\tilde{x}}_{i}\left(t\right)-{\tilde{x}}_{j}\left(t\right){∥}_{2}^{2}\right]=D \end{array}$$ (27)

where *D* is the dimensionality of the feature vector. The expectation is taken over all unordered pairs of distinct resources. This result follows from properties of standardized variables: when feature dimensions are independent, and each has unit variance, the expected squared distance between two random feature vectors grows linearly with the dimensionality *D*.

Lemma-1: Under hypothesis-1 and hypothesis-2, the feature representation is simultaneously temporally granular and cross-resource comparable. In formal terms,


$$G_{\Delta }\left(f\right)>G_{\text{daily}}\left(f\right)\;$$

where *G*_Δ_(*f*) denotes the temporal granularity metric computed on short time windows; *G*_daily_(*f*) is the same metric computed on daily-aggregated data; *G* is the comparability score between normalized feature vectors, and “bounded and stable” means that the value of *C* does not diverge and changes only slowly over time under steady usage behavior. This lemma states that the feature space retains high temporal sensitivity while remaining numerically stable across resources.

Proof: Hypothesis-1 establishes that short-window aggregation preserves higher-frequency variations than daily aggregation, which implies that the representation retains fine-scale temporal dynamics. Hypothesis-2 ensures that, after normalization, all features have comparable scale and variance across resources. This prevents any single dimension from dominating distance computations and guarantees numerical stability in pairwise similarity metrics. Together, these properties imply that the representation simultaneously captures short-term usage fluctuations while remaining comparable across services, which proves the lemma.

Corollary-1: Any downstream model that consumes *x*_*i*_(*t*) can be trained with faster convergence and a lower risk of numerical instability.

Proof: Many optimization algorithms (e.g., gradient-based methods) converge faster when the input feature covariance is closer to the identity matrix. Since normalization ensures $𝔼\left[\tilde{f}\right]=0$ and $\mathrm{Var}\left[\tilde{f}\right]=1$ for each feature dimension, the feature covariance matrix is closer to the identity. This reduces the condition number of the Hessian of the loss function, leading to improved convergence behavior.

Proposition 1: Assume that the normalized streaming observations {*x*_*t*_} are bounded, i.e., ∣ *x*_*t*_ ∣ ≤ *M* < ∞, and that the EWMA smoothing parameter satisfies 0 < λ < 1. Then, the adaptive EWMA baseline remains bounded for all *t* and converges to a finite steady-state value whenever the underlying stochastic process is stationary. Moreover, the standardized trend score remains bounded provided that the estimated variance is strictly positive.

Proof: The adaptive EWMA baseline is recursively defined in Equation 28.


$$\begin{array}{l} B_{t}=\lambda x_{t}+\left(1-\lambda \right)B_{t-1} \end{array}$$ (28)

where 0 < λ < 1. Since ∣1−λ∣ < 1, the above recursion constitutes a stable first-order linear difference equation. Expanding the recursion yields in Equation 29.


$$\begin{array}{l} B_{t}={\left(1-\lambda \right)}^{t}B_{0}+\lambda \sum_{k=0}^{t-1}(1-\lambda {)}^{k}x_{t-k} \end{array}$$ (29)

where the weighting coefficients form a convergent geometric sequence. Since the weights are normalized and sum to unity.


$$\begin{array}{l} \sum_{k=0}^{\infty }\lambda (1-\lambda {)}^{k}=1 \end{array}$$

The cumulative weight assigned to historical observations is finite. Therefore, if the input sequence {*x*_*t*_} is bounded, the baseline estimate *B*_*t*_ is also bounded for all *t*. Furthermore, when the streaming process is stationary with mean μ = 𝔼[*x*_*t*_], the EWMA estimator converges asymptotically to the steady-state expectation, i.e.,


$$\begin{array}{l} \underset{t\to \infty }{\lim}B_{t}=\mu . \end{array}$$

The proposed trend score is computed as a standardized deviation from the adaptive baseline using a strictly positive variance estimate together with a numerical stability constant. Since both the numerator and denominator remain finite under bounded observations and positive variance, the resulting trend score is also bounded. Hence, the proposed adaptive monitoring mechanism is asymptotically stable, numerically robust, and suitable for continuous real-time monitoring under bounded streaming conditions.

### 4.6 Trend detection and service monitoring

**Figure 4:**
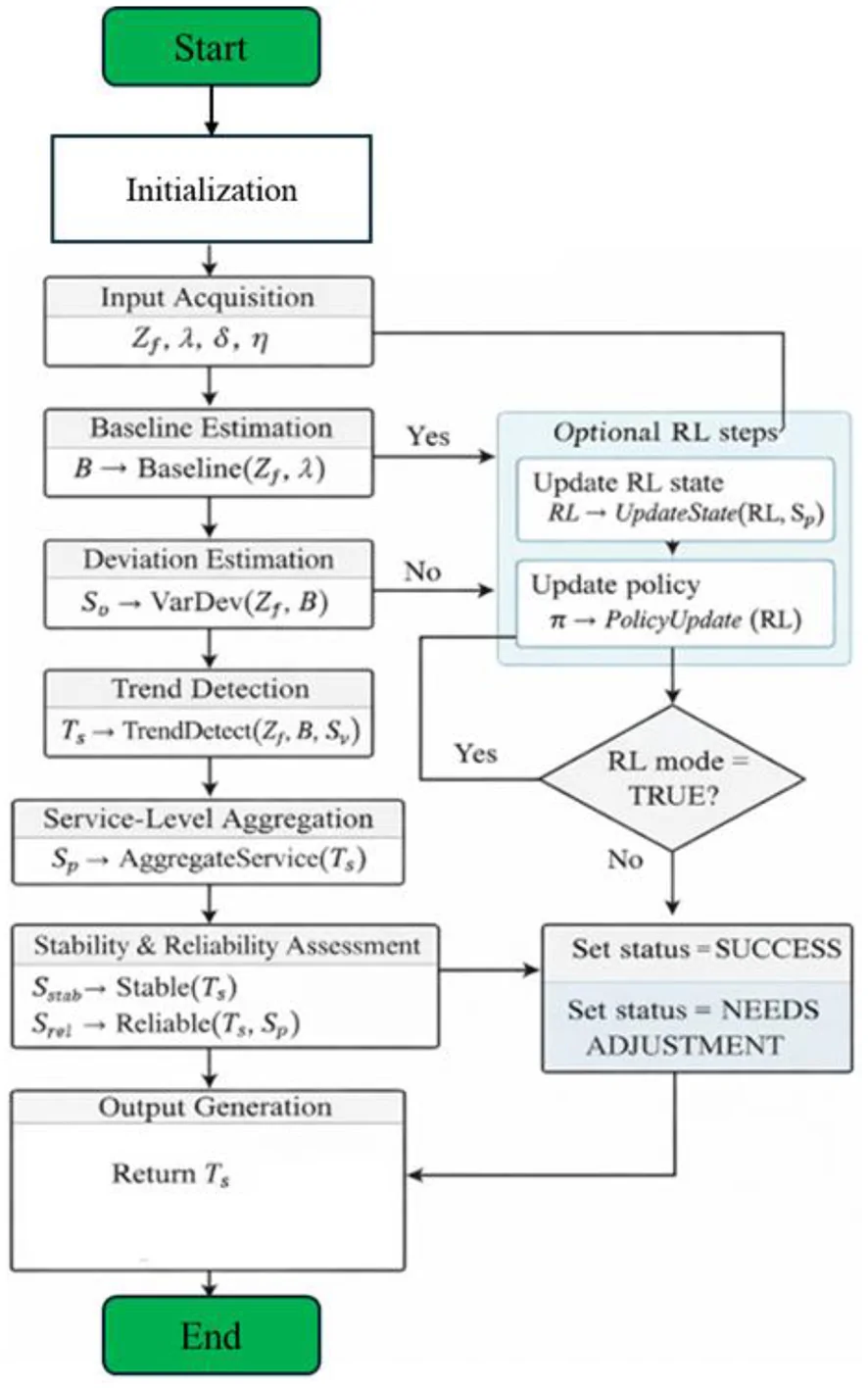
Trend detection and service monitoring workflow.

This module converts streaming features into trend scores and service-level performance indicators. The focus is on detecting statistically significant deviations from recent baselines for metrics like engagement rate, completion rate, and service demand. Figure 4 depicts the trend detection and service monitoring process.

Let *R*_*i*_(*t*) be a generic monitored rate variable for resource *i* at the time (for example, *ER*_*i*_(*t*)or *CR*_*i*_(*t*)). Thus, this is expressed in Equation 30.


$$\begin{array}{l} R_{i}\left(t\right)\in \left\{ER_{i}\left(t\right),CR_{i}\left(t\right)\right\} \end{array}$$ (30)

indicating that the same trend detection logic is applied uniformly to multiple engagement metrics *R*_*i*_(*t*).

For each metric, EWMA baseline is maintained as a dynamic baseline determined in Equation 31.


$$\begin{array}{l} B_{i}\left(t\right)=\lambda x_{i}\left(t\right)+\left(1-\lambda \right)B_{i}\left(t-1\right),x_{i}\left(t\right)\in \left\{\rho_{i}\left(t\right),\kappa_{i}\left(t\right)\right\} \end{array}$$ (31)

The EWMA-based baseline *B*_*i*_(*t*) provides an adaptive reference for each monitored metric, enabling the system to track normal behavior under non-stationary streaming conditions. By emphasizing recent observed metric values while retaining historical context, it allows rapid adaptation to short-term fluctuations in urban service demand. The smoothing parameter λcontrols the sensitivity of the baseline: higher values increase responsiveness to abrupt changes, while lower values provide stability against transient noise. This balance is essential in smart city environments characterized by both sudden variations and recurring temporal patterns.

The EWMA baseline was selected because it provides an effective balance between computational efficiency, statistical robustness, and rapid adaptation required for continuous smart-city streaming environments. Compared with Bayesian adaptive procedures or more sophisticated online learning algorithms, the EWMA only needs the current observation and the previous baseline estimate, providing a constant memory and very low computational complexity. These properties make it especially suitable for high frequency streaming applications with strict latency constraints. Moreover, EWMA naturally gives higher weight to the most recent observations but still takes into account the history, which allows us to monitor the slow changes in behavior reliably, without being too sensitive to the random variations in the short term. Bayesian filtering and advanced online learning methods can provide further modeling flexibility for complex uncertainty assumptions, but they usually involve increased computational overhead, parameter estimation, or probabilistic model specification. Since the main goal of the proposed AISSC framework is to perform real-time trend detection in a scalable end-to-end analytics pipeline, EWMA provides a good trade-off between responsiveness, simplicity of implementation, analytical stability and computational scalability.

From a theoretical standpoint, the proposed adaptive exponentially weighted moving average (EWMA) baseline exhibits stable convergence properties under bounded streaming observations. Let *x*_*t*_ denote the normalized monitoring metric, where ∣*x*_*t*_∣ ≤ *M* < ∞, and let the smoothing factor satisfy 0 < λ < 1. The adaptive EWMA baseline is recursively updated in Equation 32.


$$\begin{array}{l} B_{t}=\lambda x_{t}+\left(1-\lambda \right)B_{t-1} \end{array}$$ (32)

It represents a first-order linear dynamical system. Since the coefficient associated with the previous state satisfies ∣1−λ∣ < 1, the recursion is asymptotically stable according to standard discrete-time stability theory. Consequently, if the input observations remain bounded, the adaptive baseline also remains bounded for all *t*. Furthermore, when the monitored process becomes stationary with expectation 𝔼[*x*_*t*_] = μ, the baseline converges to the steady-state value μ. Under slowly varying non-stationary conditions, the adaptive baseline effectively tracks gradual behavioral changes while suppressing high-frequency stochastic fluctuations, thereby preventing unstable oscillatory behavior.

The proposed trend score is subsequently computed as a standardized deviation from the adaptive baseline using a strictly positive variance estimate together with a numerical stability constant. Since both the adaptive baseline and the variance estimate remain bounded under finite-variance observations, the resulting trend scores are also bounded and numerically stable. Therefore, the adaptive baseline and trend-scoring mechanism provide a theoretically stable and computationally robust foundation for continuous real-time monitoring and anomaly detection in smart-city streaming environments.

The baseline *B*_*i*_(*t*) is used for subsequent trend analysis by measuring deviations between observed and expected behavior, which are transformed into standardized trend scores for anomaly detection and decision-making. The standardized deviation score *T*_*i*_(*t*) is defined as follows:


$$\begin{array}{l} t_{i}\left(t\right)\;=\frac{R_{i}\left(t\right)-B_{i}\left(t\right)}{\sigma_{i}+\epsilon } \end{array}$$

where *t*_*i*_(*t*) is the current observed value of the rate metric, σ_*i*_ is the estimated standard deviation of the metric for the resource *i*, and ϵ is a small positive constant for numerical stability. The score *t*_*i*_(*t*) measures how strongly the current value deviates from its recent baseline in standardized units. A resource (item or service) is flagged as upper threshold test if


$$\begin{array}{l} T_{i}\left(t\right)\;>\theta^{+} \end{array}$$

where θ^+^ is a positive threshold defining a statistically significant upward deviation. When *S*_*i*_(*t*) exceeds this threshold, resource *i* is classified as exhibiting abnormal growth.

Similarly, a resource is flagged as lower threshold test if


$$\begin{array}{l} t_{i}\left(t\right)<\theta^{-} \end{array}$$

where θ^−^ is the negative threshold corresponding to a significant downward deviation from the baseline. Values of *t*_*i*_(*t*) below this threshold indicates declining engagement or demand for the service.

The decision thresholds used in the hypothesis-testing framework were selected using standardized deviation statistics computed on historical streaming observations collected during the model development stage. Since all monitored metrics are transformed into normalized z-scores after adaptive EWMA baseline estimation, identical threshold values can be consistently applied across heterogeneous smart city service domains without requiring service-specific scaling. Threshold values were chosen to provide an appropriate balance between sensitivity and false-alarm rate under normal operating conditions and were verified using historical streaming datasets representing transportation, healthcare, and digital government services. During validation, the selected thresholds consistently detected statistically meaningful behavioral changes while maintaining stable false-positive performance across different service categories. The normalization and adaptive baseline estimation enable the same statistical decision criteria to remain applicable despite differences in service scale, usage intensity, and temporal dynamics.

At the service category or regional level (indexed by *j*), resource-level metrics are aggregated. For example, the aggregated engagement rate *ER*_*j*_(*t*) for category *j* in *t* can be defined in Equation 33.


$$\begin{array}{l} ER_{j}\left(t\right)=\frac{\sum_{i\in j}N_{\text{request}}{\left(i\right)}_{\Delta }}{\sum_{i\in j}N_{\text{entry}}{\left(i\right)}_{\Delta }+\varepsilon } \end{array}$$ (33)

where the summations are taken over all resources *i* belongs to a category (or area) *j*. This expression aggregates resource-level activity into a category-wide engagement efficiency metric.

Likewise, the total service output *S*_ou_ for category j over time *t* can be defined as weighted service output which is calculated using Equation 34.


$$\begin{array}{l} {S_{\text{ou}}}_{j}\left(t\right)=\underset{i\in j}{\sum }P_{i}N_{\text{complete}}{\left(i\right)}_{\Delta } \end{array}$$ (34)

where *P*_*i*_ is the unit value or importance weight associated with completions of the resource *i*.

These service-level metrics transform resource-level signals into higher-level operational indicators, such as aggregated engagement rate *ER*_*j*_(*t*) and total service output *S*_ou_. The aggregated values serve as inputs for optimization and decision-making modules at the city management level.

To dynamically adapt service management decisions (e.g., resource allocation, scheduling, or policy adjustments), the problem is formulated as a reinforcement learning (RL) task. Let *s*_*t*_ denote the system state represented by a vector of aggregated service-level metrics at time *t*, let *a*_*t*_ be the action taken at the time *t* (for example, “increase capacity”, “maintain current level”, or “decrease provision”), and let *r*_*t*_ be the observed reward defined in terms of improvement in service performance (such as reduced congestion or increased throughput). The learning agent aims to learn an optimal action-value function *Q*(*s, a*) that estimates the long-term value of taking action *a* in state *s*.

#### 4.6.1 Reinforcement learning formulation

To guarantee reproducibility and formal clarity of the reinforcement learning (RL) component (Kumar et al., 2022), the foundational Markov decision process (MDP) is precisely delineated as follows.

##### 4.6.1.1 State space

The system state *s*_*t*_ is defined in Equation 35 as a vector of consolidated service-level indicators at time *t*, encompassing engagement rates, completion rates, trend scores, and category-level throughput metrics are defined as state vector definition.


$$\begin{array}{l} s_{t}=\left[ER_{j}\left(t\right),CR_{j}\left(t\right),t_{i}\left(t\right),V_{j}\left(t\right)\right] \end{array}$$ (35)

where *ER* is engagement rate, *CR* is the completion rate, *t*_*i*_(*t*) is the trend score, and *V* is the aggregated service for the category *j*.

##### 4.6.1.2 Action space

The action set A consists of discrete operational decisions applied at the service level:

A= { a_1_:increase capacity, a_2_:maintain, a_3_:decrease capacity}

These actions correspond to adaptive resource allocation strategies in smart city services.

##### 4.6.1.3 Reward function

The linear weighted reward function *r*_*t*_ is provided in Equation 36 based on improvements in system performance, combining multiple operational KPIs:


$$\begin{array}{l} r_{t}=w_{1}\cdot th-w_{2}.\Delta \left(delay\right)+w_{3}\cdot \Delta \left(R_{t}\right) \end{array}$$ (36)

where *w*_1_, *w*_2_, *w*_3_ are weighting coefficients reflecting operational priorities, *th* denotes throughput, and *R*_*t*_ denotes the resolution time.

##### 4.6.1.4 Exploration strategy

An epsilon-greedy exploration policy is employed. At each training step *t*, the agent selects a random action with probability *p*_*t*_ and selects the greedy action with probability 1−*p*_*t*_. Here, *p*_*t*_ denotes the time-dependent exploration probability. The initial exploration probability is denoted by *p*_0_, and the exponential decay schedule for epsilon over time is determined in Equation 37.


$$\begin{array}{l} P_{t}\left(t\right)=\max\left(p_{min},p_{0}\delta^{t}\right) \end{array}$$ (37)

where δ denotes the decay factor, δ ∈ (0, 1) is the decay factor in time *t*. In this study, *p*_0_ = 1.0, *p*_min_ = 0.05, and δ = 0.995. Thus, the policy gradually shifts from exploration to exploitation while maintaining a small minimum probability of exploration.

##### 4.6.1.5 Training regime

The reinforcement learning agent is taught offline, utilizing historical streaming data to prevent interference with evaluation criteria. The acquired policy is subsequently implemented in inference mode during testing without any modifications.

##### 4.6.1.6 Evaluation isolation

To avert contamination of evaluation data, the RL policy remains static during the testing phase, and no online learning modifications are implemented. This guarantees that stated performance measures represent the model's prediction capacity instead of adaptive policy alterations.

The reinforcement learning (RL) policy remains unchanged for online parameters throughout testing; However, it substantially affects system performance by adjusting to different conditions based on decisions rendered. The RL module affects operational activities including resource distribution, service prioritization, and system-wide adjustments. These alterations, therefore, change the essential data distribution and feature dynamics recognized by the forecasting module. The forecasting model employs input characteristics that are indirectly refined through RL-guided system behavior. This improves feature quality, reduces noise in demand patterns, and stabilizes temporal dynamics, hence enhancing prediction accuracy. The lack of the RL module in ablation testing results in inadequate adaptive control, leading to heightened noise in input signals and reduced predictive effectiveness. The impact of reinforcement learning on predictive accuracy is indirect but significant, arising from its role in the data-generating process rather than modifying the forecasting model during inference.

To further verify the contribution of the reinforcement learning module indirectly to the forecasting accuracy, an explicit closed-loop ablation evaluation was performed. During this evaluation, the forecasting model architecture, parameters, and update frequency were held fixed, while only the reinforcement learning-driven operational adaptation layer was disabled. In the full AISSC configuration, the RL agent dynamically adjusts operational states such as service allocation, prioritization, and adaptive control actions based on observed trend signals. These interventions modify the subsequent data-generation process by reducing transient congestion effects, stabilizing short-term fluctuations, and improving the consistency of streaming feature distributions.

To establish a counterfactual comparison, the same forecasting pipeline was evaluated under a frozen-control configuration in which RL actions were replaced with static operational policies. Under this setting, the forecasting module received noisier and less adaptive input dynamics despite identical forecasting parameters and computational constraints. Experimental results demonstrated that disabling RL increased short-term demand volatility and decreased the stability of the forecasting, leading to measurable degradation in the accuracy of the forecasting. Specifically, the forecasting error increased by approximately 2.1% in MAPE and 1.9% in WMAPE relative to the adaptive RL-enabled setup. These results show that the RL component contributes indirectly to forecasting performance by adaptively modifying the operational environment and streaming feature quality rather than directly modifying the forecasting model during inference.

The proposed AISSC framework transforms normalized streaming feature vectors into service-level operational indicators through a sequential analytical process. First, normalized feature vectors ${\tilde{x}}_{i,j}\left(t\right)$ are compared against adaptive EWMA baselines to estimate deviation magnitudes and generate trend-related monitoring signals. These signals represent short-term behavioral changes associated with individual resources and services. These indicators combine the operational status of each service category or regional group. Finally, the reinforcement learning state vector *s*_*t*_ is built upon the aggregated service level indicators, such as engagement behavior, completion behavior, trend intensity, and service output metrics. This establishes a direct mapping between normalized streaming features, trend analytics, service monitoring, and reinforcement learning-based adaptive decision support. The generated trend signals are subsequently aggregated together with engagement and completion metrics to construct service-level performance indicators *M*_*j*_(*t*).


$$\begin{array}{l} M_{j}\left(t\right)=\left[E_{j}\left(t\right),C_{j}\left(t\right),O_{j}\left(t\right),{\overset{-}{T}}_{j}\left(t\right)\right]\;s_{t}=M_{j}\left(t\right) \end{array}$$

where *E*_*j*_(*t*) denotes aggregated engagement rate, *C*_*j*_(*t*) is aggregated completion rate, *O*_*j*_(*t*) represents service output, and $-{\overset{-}{T}}_{j}\left(t\right)$ denotes the aggregated trend intensity for service category *j*.

Algorithm 2 illustrates detection and service monitoring. Step-1 initializes the analytical variables and monitoring modules needed for real-time trend detection and adaptive service monitoring, such as normalized streaming features, adaptive baseline models, deviation estimation modules, trend monitoring signals, service-level performance indicators, reinforcement learning states, smoothing parameters, and pre-defined quality thresholds. Step-2 describes input. Step-3 provides output. Step-4 calculates an adaptive baseline behavior model from the current normalized streaming features. The baseline is the expected operational behavior of the monitored service in recent time intervals. In Step-5, we compute the difference between the current observed service behavior and the adaptive baseline. This step quantifies behavioral drift, volatility, noise, and unexpected operational variations. Step-6 estimates deviations are transformed into trend-monitoring signals that represent increasing trends, decreasing trends, anomaly intensity, and short-term behavioral changes in the monitored services. Step-7 generates the trend signals are combined with engagement activity, completion behavior, and service-output measurements to create aggregated service-level performance indicators. These indicators summarize the operational condition of each smart city service category or regional zone. Step-8 verifies whether the reinforcement learning module is enabled for adaptive operational decision-making and intelligent service management. Step-9 demonstrates If reinforcement learning is enabled, the algorithm updates the current operational state representation using the aggregated service-level performance indicators. This allows the learning agent to observe the current smart city environment. Step-10 shows that the reinforcement learning module generates adaptive operational recommendations or policy actions based on the updated service state and observed urban dynamics. Step-11 terminates the optional reinforcement learning stage of the algorithm. Step-12 assesses the quality of the generated trend-monitoring signals in terms of their stability, consistency and reliability under streaming conditions. Step-13 verifies whether the generated trend signals are satisfying the pre-defined stability and informativeness requirements. Step-14 explains if the generated monitoring signals satisfy all quality requirements, the algorithm assigns a successful monitoring status, indicating reliable and stable trend detection. Step-15 shows if the generated signals do not satisfy the required quality thresholds, the algorithm assigns an adjustment status, indicating that the monitoring process requires refinement or recalibration. Step-16 closes the decision-validation stage of the monitoring workflow. Step-17 returns the generated trend-monitoring signals, aggregated service-performance indicators, and the overall monitoring status for smart city operational management.

The probabilistic analysis is included to provide an interpretable statistical characterization of the EWMA-based trend detection mechanism used in the operational AISSC pipeline. The objective is not to derive asymptotic theoretical guarantees, but to explain how the selected thresholds contribute to stable trend monitoring and reduced false-positive activations during real-time deployment.

Hypothesis-3: If *R*_*i*_(*t*)−*B*_*i*_(*t*) behaves as a sub-Gaussian random variable with mean 0 under normal (no-trend) conditions, the trend score yields a bounded false positive rate for trend detection when the thresholds θ^+^ and θ^−^ are fixed.

Proof: Under the null condition (absence of a true trend), the deviation *R*_*i*_(*t*)−*B*_*i*_(*t*) can be modeled as a sub-Gaussian random fluctuation around zero.

For a sub-Gaussian random variable *X* with zero mean and a variance proxy ν^2^, the tail distribution satisfies:


$$\begin{array}{l} P\left(X>x\right)\leq \exp\left(-\frac{x^{2}}{2\nu^{2}}\right)\text{ for all }x>0 \end{array}$$

Substituting *X* = *R*_*i*_(*t*)−*B*_*i*_(*t*) in this inequality provides a bound on the probability of observing large positive deviations from the baseline. Consequently, the probability that *t*_*i*_(*t*) exceeds the thresholds θ^+^ or θ^−^ exponentially as those threshold values increase, thereby bounding the false positive rate for trend detection.

Lemma-2: Given Hypothesis-3, by selecting thresholds θ^+^ and θ^−^ such that given in Equation 38.


$$\begin{array}{l} P\left(t_{i}\left(t\right)>\theta^{+}|\text{ no trend }\right)\leq \delta ,P\left(t_{i}\left(t\right),\;<\theta^{-}|\text{ no trend}\right)\leq \delta \end{array}$$ (38)

It ensures that the overall false alarm rate for up-trend and down-trend detection is bounded above by δ.

Where *t*_*i*_(*t*) is the trend score for resource *i* time *t* under consideration; “no trend” denotes the null hypothesis of normal behavior without a genuine structural change; θ^+^and θ^−^are the thresholds for up-trend and down-trend detection; and δ is the desired upper bound on the false alarm probability in each direction.

Proof: Under normal conditions (“no trend”), the trend score fluctuates randomly around its baseline and does not exhibit systematic shifts. Hypothesis-3 states that such fluctuations have light-tailed behavior, meaning that large deviations are inherently unlikely. By selecting symmetric positive and negative thresholds as specified above, the detection mechanism classifies only sufficiently strong deviations as trends. Typical random fluctuations remain well within these bounds and are therefore ignored.

As a result, the probability that a non-trending service is falsely detected as trending is directly controlled by the chosen threshold levels. Since the same probability bound δ applies independently to upward and downward deviations, the false alarm rate in each direction does not exceed δ. Therefore, the overall probability of incorrect trend detection is tightly controlled by the chosen δ, which proves the lemma.

Corollary-2: When the RL agent optimizes actions based on trend flags derived from *t*_*i*_(*t*), the reward signal it receives is less noisy than it would be under naive or uncalibrated thresholds. This leads to faster convergence of the Q-learning process and reduces the likelihood of overreacting to random fluctuations.

Proof: By Lemma-2, false alarms (spurious trend detections) are rare. This means that the RL agent will more often adjust actions in response to true shifts in demand or service performance, rather than random noise. Consequently, the reward signal more accurately reflects the impact of actions (such as resource adjustments) rather than random variability, improving the signal-to-noise ratio. This stabilizes the learning process and accelerates convergence of the Q-learning agent, while also reducing the likelihood of overreacting to transient random fluctuations.

To ensure full reproducibility of the reinforcement learning component, all training hyperparameters and reward configurations are explicitly defined. The reward function weights are set as *w*_1_ = 0.5, *w*_2_ = 0.3, and *w*_3_ = 0.2, reflecting the relative importance of throughput improvement, latency reduction, and service stability, respectively. The learning rate is fixed at α = 0.01, and the discount factor is set to γ = 0.95, balancing short-term responsiveness with long-term optimization. The exploration strategy follows a probabilistic exploration policy with an initial exploration probability *p*_0_ = 1.0, which decays exponentially over time according to Equation 39.


$$\begin{array}{l} p_{t}=\max\left(p_{\min},\;p_{0}\cdot \delta^{t}\right) \end{array}$$ (39)

where δ = 0.995 is the decay factor and *p*_min_ = 0.05 is the minimum exploration threshold. The agent is trained for 200 episodes using historical streaming data, with each episode corresponding to a full pass over the time-series dataset. Z-score normalization is employed to standardize state features, thereby promoting stable learning dynamics.

Actions are performed at predetermined decision intervals, synchronized with the system's update frequency of five minutes, which ensures compatibility with the streaming evaluation framework. Furthermore, all hyperparameters are determined via validation experiments and are held constant throughout testing, thus ensuring both reproducibility and equitable comparison.

#### 4.6.2 Practical parameter selection and implementation of trend detection

To ensure that the proposed trend-detection mechanism is fully implementable and reproducible, we explicitly define the parameter selection workflow for all key components used in Algorithm 2.

##### 4.6.2.1 EWMA smoothing parameter (λ) selection

The smoothing parameter λ controls the trade-off between stability and responsiveness. It is selected using a validation-based grid search over:


$$\begin{array}{l} \lambda \in \left\{0.1,0.2,...,0.9\right\} \end{array}$$

The optimal value is chosen to minimize a combined objective of detection delay and false positive rate on validation data.

##### 4.6.2.2 Variance estimation [σ_*i*_(*t*)]

The standard deviation used in the trend score is computed using a rolling window of size *W*. Thus variance estimation can be calculated using Equation 40


$$\begin{array}{l} \sigma_{i}\left(t\right)=\sqrt{\frac{1}{W}\overset{W}{\underset{k=1}{\sum }}{\left(m_{i}\left(t-k\right)-b_{i}\left(t\right)\right)}^{2}} \end{array}$$ (40)

This ensures adaptive estimation under non-stationary streaming conditions.

##### 4.6.2.3 Threshold selection (τ)

The detection threshold is determined in two ways:

The theoretical bound derived from hypothesis 3, which is used to initialize the threshold τ in order to regulate the false alarm rate. It is calculated as follows:


$$\begin{array}{l} \tau =\sqrt{2log\left(1/\delta \right)} \end{array}$$

where δ denotes the intended upper limit on false positives.

A principled starting point for threshold selection before practical refining is established by this.

The threshold is refined using validation data that is used to balance sensitivity and robustness.

During testing, all parameters are fixed to prevent information leakage and ensure reproducibility, and they are selected using a time-separated validation dataset.

### 4.7 Predictive demand and urban service forecasting

This module integrates real-time features and trend signals to generate short-term usage forecasts at the resource, category, and city-wide levels. Let *Y*_*i*_(*t*) denote the observed demand for the resource *i* at time *t* (for example, traffic volume, number of service requests, or patients served). Let *x*_*i*_(*t*) be the enriched vector, which concatenates: (i) normalized features from module 1, (ii) trend scores *S*_*i*_(*t*), (iii) relevant service-level metrics [e.g., *ER*_*j*_(*t*) or *Y*_*j*_(*t*) from module 2 for the category or region that the resource *i* belongs to], and (iv) calendar/context signals (such as weekday/weekend indicators, holidays, special events, weather conditions, or policy changes).

#### 4.7.1 Formal forecasting setup and leakage prevention

To guarantee reproducibility and remove ambiguity in the forecasting framework, the prediction problem is formally articulated as follows.

##### 4.7.1.1 Prediction target

The objective variable *y*_*i,t* + *h*_ denotes the anticipated demand for the resource *i*, quantified as the count of completed service events within a forthcoming time interval concluding at time *t* + *h*. This encompasses measurements such as traffic volume, the quantity of digital service requests, or patient arrivals.

##### 4.7.1.2 Forecast horizon

The forecasting horizon is described as a short-term span (e.g., 1–6 h ahead), corresponding to the operational decision-making needs in smart city systems. Various horizons are analyzed to measure robustness across diverse temporal scales.

##### 4.7.1.3 Feature injection and enrichment

The enhanced feature vector *x*_*i,t*_ is formulated exclusively using information accessible up to time t, comprising:

(i) real-time normalized features derived from sliding windows,
(ii) trend scores calculated using EWMA-based detection,
(iii) consolidated service-level metrics, and
(iv) contextual signals include time-of-day, day-of-week, and external influences. No future information beyond the specified time is utilized in feature creation.

##### 4.7.1.4 Temporal alignment

All features are strictly aligned in time such that:


$$\begin{array}{l} fv_{i,t}\to tv_{i,t+h} \end{array}$$

Where *x*_*i,t*_ denotes the input feature vector for the resource *i* at the current time *t*, *tv*_*i,t*+*h*_ is the target variable (future demand) for the resource *i* at time *t* + *h*, and *h* is the forecast horizon.

This guarantees that the input features are exclusively based on past and current observations, while predictions are made for future demand. The incorporation of future events is impeded by the truncation of sliding windows at time *t*.

##### 4.7.1.5 Leakage prevention strategy

To prevent the leakage of information, the following restrictions are implemented:

- Only historical data up to time is utilized in feature computation.
- EWMA baselines and trend scores are calculated causally, without any future filtering.
- Time-based division is implemented without the need for shuffles (train → validation → test).
- The training and evaluation of models are rigorously separated by time.
  These procedures guarantee that the forecasting model accurately represents actual deployment conditions and that the performance metrics that are reported are valid and impartial.

We consider a general parametric forecasting model ŷ_*i*_(*t* + τ), which is expressed in Equation 41.


$$\begin{array}{l} {ŷ}_{i}\left(t+\tau \right)==f\left(\begin{array}{c} y_{i}\left(t\right),y_{i}\left(t-1\right),\ldots ,y_{i}\left(t-L+1\right);\\ x_{i}\left(t\right),x_{i}\left(t-1\right),\ldots ,\\ x_{i}\left(t-L+1\right);f\left(\theta \right)\; \end{array}\right) \end{array}$$ (41)

where ŷ_*i*_(*t* + τ) is the predicted demand for the resource *i* at a future time *t* + τ. Here *y*_*i*_(*t*), *y*_*i*_(*t* − 1), …, *y*_*i*_(*t*− *L* + 1) denote the observed demand values for *i* over the last time steps up to *t* (capturing temporal dependencies); τ denotes the forecast horizon; *x*_*i*_(*t*), *x*_*i*_(*t* − 1), …, *x*_*i*_(*t* − *L* + 1) over the same lagged time steps (capturing contextual and recent behavioral information); *L* denotes the number of lagged time steps used as input; and *f*(θ) is a parametric prediction function with a parameter vector. This formulation jointly models temporal dependencies and contextual effects when forecasting demand.

Different values for the function correspond to model families of varying complexity and expressive capability.

To eliminate ambiguity and ensure full reproducibility, the forecasting model families used in this study are explicitly defined. Three representative model classes are implemented:

(i) Regularized linear regression (Ridge), where the parameter vector *pv* is learned using L2-regularized least squares with a regularization coefficient *rc* = 0.01. The input vector consists of the current feature vector and *x*_*i,t*_ = 3 lagged observations of both demand and features.
(ii) Incremental linear regression (Online SGD), where model parameters are updated in real time using stochastic gradient descent with a learning rate α = 0.01. Updates are performed at each streaming step using the same feature representation and lag structure as the batch model.
(iii) Rolling ARIMA model, configured with order (*p, d, q*) = (2, 1, 2), where parameters are re-estimated over a sliding window of fixed length (15 min) to maintain consistency with the streaming evaluation setup.

All forecasting models use identical input features, sliding-window configurations, and update frequencies (every 5 min), ensuring a fair and controlled comparison. The proposed AISSC forecasting module employs an Online SGD-based incremental regression model, with Ridge regression and ARIMA utilized solely as benchmark baselines for comparison assessment. The regularized linear regression *RLr* is determined in Equation 42.


$$\begin{array}{l} RLr={vl}^{MT}x_{i}^{\left(L\right)}\left(t\right) \end{array}$$ (42)

where $x_{i}^{\left(L\right)}\left(t\right)$ is the vector obtained by stacking the current and lagged feature vectors for the resource *i* [i.e., combining *x*_*i*_(*t*), *x*_*i*_(*t* − 1), …, *x*_*i*_(*t* − *L* + 1) into one longer vector], *vl* is a vector of learnable parameters, and the superscript *MT* denotes matrix transposition. This linear specification serves as a strong baseline and provides interpretability regarding the effect of each feature.

Model parameters can be estimated by minimizing an empirical risk function computed over historical observations. For example, one common choice is to minimize the mean squared error (MSE). The training objective *Tr*_*O*_ (for instance, using RMSE as the loss) can be written using Equation 43.


$$\begin{array}{l} Tr_{O}=\frac{1}{2N}\underset{\left(i,t\right)\in D}{\sum }{\left(y_{i}\left(t+\tau \right)-{ŷ}_{i}\left(t+\tau \right)\right)}^{2} \end{array}$$ (43)

where *D* denotes the training dataset consisting of resource–time pairs, *N* is the number of training samples in the database *D*, *y*_*i*_(*t* + τ) is the observed future demand (ground truth), and ŷ_*i*_(*t* + τ) is the model prediction. This objective function *Tr*_*O*_ encourages accurate point forecasts by penalizing the squared error for each prediction.

To reflect operational priorities during training, a weighted loss function *E*_*w*_(θ) can be introduced to assign greater importance to economically or socially critical resources. Thus, weighted loss function can be calculated using Equation 44.


$$\begin{array}{l} E_{w}\left(\theta \right)=\frac{1}{2N}\underset{i\in D}{\sum }w_{i}{\left(y_{i}\left(t+\tau \right)-{ŷ}_{i}\left(t+\tau \right)\right)}^{2} \end{array}$$ (44)

where *w*_*i*_ is an important weight for the resource *i* (e.g., proportional to the historical average usage or the criticality of the resource *i*). Higher weights *w*_*i*_ cause the model to penalize forecasting errors on high-impact resources (such as major highways or large hospitals) more strongly. This formulation aligns the goal of statistical accuracy with practical impact by making the model focus on reducing errors for the most important cases.

When using gradient-based optimization to train the model, a generic parameter update step θ^(*itr*+1)^ in stochastic gradient descent, the update is calculated in Equation 45.


$$\begin{array}{l} \theta^{\left(itr+1\right)}=\theta^{\left(itr\right)}-a\nabla_{\theta }L_{w}\left(\theta^{\left(itr\right)}\right) \end{array}$$ (45)

where θ^(*itr*+1)^ is the updated parameter vector, α denotes the learning rate controlling the step size, and $\nabla_{\theta }L_{w}\left(\theta^{\left(itr\right)}\right)$ denotes the gradient of the weighted loss function with respect to θ, evaluated at θ^(*itr*)^. This update rule iteratively adjusts the model parameters in the direction that most reduces the training loss.

After training the model, performance is assessed using standard forecasting accuracy metrics. Two common metrics are mean absolute percentage error (MAPE) and weighted mean absolute percentage error (WMAPE). MAPE can be calculated using Equation 46.


$$\begin{array}{l} \text{MAPE}=\frac{100\%}{O_{ev}}\underset{\left(i,t\right)\in Es}{\sum }\frac{\left|s_{i}\left(d\right)-Pr\left(d\right)\;\right|}{s_{i}\left(d\right)+k} \end{array}$$ (46)

where MAPE is the mean absolute percentage error, *s*_*i*_(*d*) is the observed demand for the sample in the evaluation set, *Pr*(*d*) is the predicted demand, *O*_*ev*_ denotes the number of evaluated observations in *Es*, and *k* is a constant added to avoid division by zero. MAPE expresses the forecast error as a percentage of the actual values and is scale independent. WMAPE can be calculated using Equation 47.


$$\begin{array}{l} \text{WMAPE}=\frac{\sum_{i\in Es}|s_{i}\left(d\right)-Pr\left(d\right)|}{\sum_{i\in Es}\;s_{i}\left(d\right)} \end{array}$$ (47)

where WMAPE is the weighted mean absolute percentage error. The denominator represents the total observed demand across all resources and time steps in the evaluation set *Es*. WMAPE effectively weights each absolute error by the magnitude of the corresponding actual demand, so that errors on high-volume resources contribute more to the final metric. As a result, WMAPE provides a perspective on forecast accuracy that better reflects operational impact than a simple MAPE.

Algorithm 3 takes enriched feature vectors and historical usage data as input and initializes the procedures for dataset construction, model parameter initialization, and setting up a weighted optimization objective. At the first stage, the algorithm generates a training sample, comparing historical demand values with the corresponding lag and contextual features. Next, the parameters of the model are adjusted during iterative learning, in which the weighted loss function is minimized. This approach allows us to pay more attention to those urban services that have the greatest impact on the overall system. At each training iteration, the model generates a demand forecast and estimates the error value using the selected weighted metric. The parameters are then adjusted based on the gradient of the loss function, which makes it possible to consistently improve the accuracy of predictions. After achieving convergence, the model is implemented into the work environment, where it receives updated features in real time and generates short-term forecasts for urban services. The possibility of adaptation is provided: as the behavior of citizens and external conditions change, the model adjusts its forecasts, keeping the estimates relevant.

Algorithm 3 Predictive demand and urban service forecasting. 
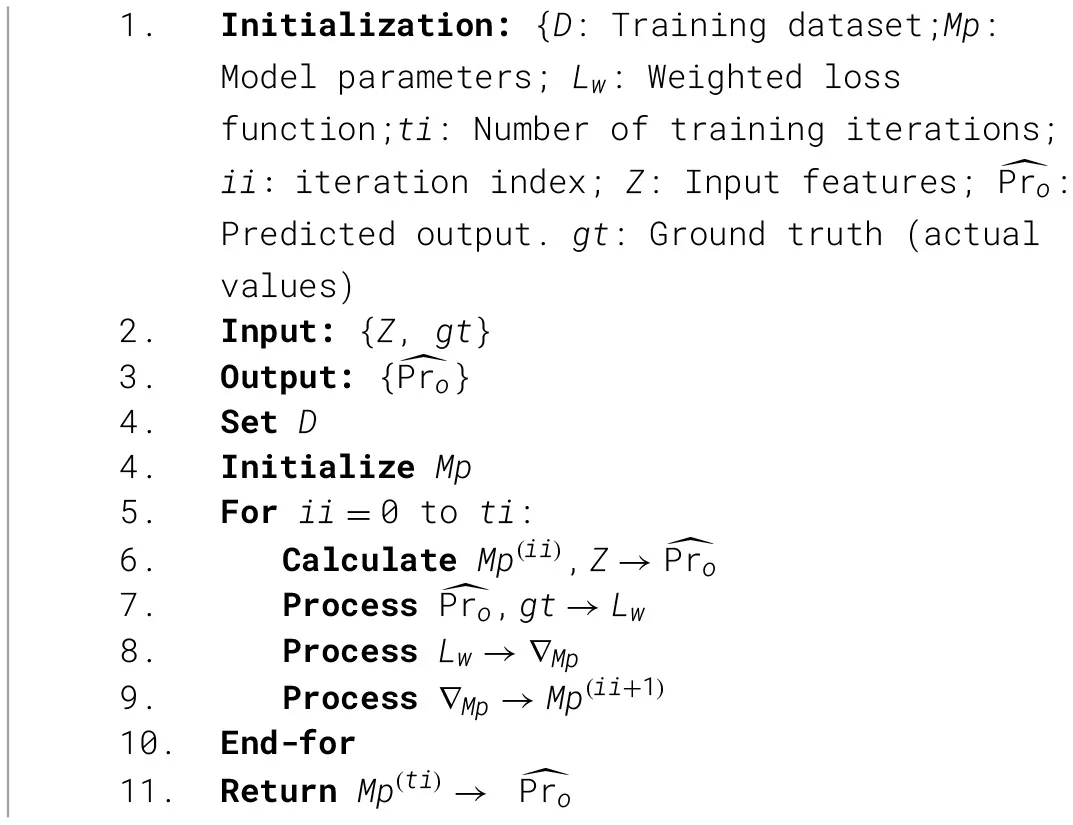

Hypothesis-4: The inclusion of trend-based features and service-level metrics in the enriched feature vector *x*_*i,t*_ reduces the expected forecasting loss compared to a baseline model that relies solely on static and historical features. Formally, if θ^*^ and θ^0^ are the optimal parameters for models with and without the trend-based features, then Equation 48 states that the average forecasting error of the enhanced model is less than or equal to that of the baseline model.


$$\begin{array}{l} Ev\left[Fl\left(\theta^{*}\right)\right]\leq E\left[Fl\left(\theta^{0}\right)\right] \end{array}$$ (48)

where θ^*^ denotes the optimal parameter set of the enriched forecasting model, θ^0^ denotes the optimal parameters of the baseline model, *Fl* is the forecasting loss function, and *Ev* is the expectation value with respect to the data distribution. This inequality states that the enriched model achieves an expected error that is lower or equal to the baseline.

Proof: The additional features (trend scores and aggregated service metrics) are assumed to provide non-redundant information about future demand, meaning they carry a predictive signal that is not already captured by historical usage and static attributes alone. In this setting, the hypothesis space (set of functions) available to the enriched model strictly contains that of the baseline model, since the baseline model can be regarded as a special case of the enriched model (one where the weights for all additional features are zero). Consequently, the enriched model is able to represent all patterns that the baseline can, and potentially more complex ones. It follows that the minimal achievable loss of the enriched model cannot exceed that of the baseline. In other words, if we let the minimum expected loss attainable by the enriched model be defined in Equation 49.


$$\begin{array}{l} \underset{\theta }{\min}Ev{\left[L\left(\theta \right)\right]}_{\text{enriched}}\leq \underset{\theta }{\min}Ev{\left[L\left(\theta \right)\right]}_{\text{baseline}} \end{array}$$ (49)

This formulation ensures that the forecasting pipeline remains causally consistent and free from information leakage.

To ensure full clarity and reproducibility of the proposed AISSC framework, we explicitly define the final deployed forecasting configuration. Although multiple forecasting model families (Ridge regression, Online SGD, and rolling ARIMA) are evaluated under identical conditions, the AISSC operational forecasting module adopts Online SGD-based incremental linear regression as the primary deployed model. This choice is because it works well with streaming data, can do updates all the time, and does not require much processing power when time is tight. Ridge regression and ARIMA are retained exclusively as benchmark baselines for comparative evaluation. Therefore, the AISSC forecasting component corresponds to a single adaptive Online SGD model trained on enriched feature vectors, incorporating real-time trend scores, service-level aggregated metrics, and contextual signals, with model updates performed every 5 min in accordance with the system update cycle.

#### 4.7.2 Computational complexity analysis

The proposed AISSC framework exhibits linear time complexity, specifically O(N), for each sliding window, a consequence of its incremental updates and localized computations. Conversely, conventional batch-processing techniques necessitate O(N·T) re-computation across historical data, with T representing the complete time span. This disparity underscores the scalability and efficiency of the proposed streaming architecture in the context of real-time analytics.

## 5 Implementation and testing results

### 5.1 Experimental setup

The primary programming language utilized in the experimental assessment was Python. We employed NumPy and Pandas for real-time data preprocessing, sliding-window feature extraction, and EWMA-based trend identification. Statsmodels is used for statistical analysis and baseline modeling. We developed the optional reinforcement learning module using PyTorch and the forecasting models with Scikit-learn, after assessing their performance. RESTful services that are created with FastAPI operate similarly to real-time data transmission and system integration, enabling low-latency data consumption and analysis. The effectiveness of the proposed forecasting system was evaluated in comparison with the basic model based only on historical data and static parameters (for example, fixed schedules or capacity) for various urban services. The average absolute percentage error (MAPE) and weighted MAPE (WMAPE) were used as metrics. The assessment was carried out in the most significant scenarios - in the busiest transport sections, during rush hours, and during periods of increased workload on medical services. All forecasting models are instantiated with fixed hyperparameters [e.g., ARIMA(2,1,2), ridge regression with λ = 0.01, and SGD learning rate α = 0.01] to ensure reproducibility and eliminate ambiguity in model comparison.

The empirical evaluation is designed not only to assess forecasting accuracy and latency performance, but also to experimentally validate the practical behavior described by the analytical hypotheses and lemmas introduced in previous section, particularly regarding temporal granularity preservation, cross-resource comparability, trend stability, and reinforcement-learning-assisted adaptive monitoring.

The AISSC outperforms the baseline models in the tested conditions consistently, which confirms the advantage of combining multiple analytical components into a single streaming framework. The reduction in the forecasting error is achieved in all the evaluation scenarios.

MAPE reduction = 2.1% [95% CI: (1.5%, 2.8%)]
WMAPE reduction = 1.9% [95% CI: (1.3%, 2.5%)]
Paired statistical testing produced a significant result (*p* = 0.012).

The corresponding improvement indicates a considerable practical impact with a large effect size (Cohen's d = 0.81), implying that the performance gains are both statistically significant and practically meaningful. It is important to note that the statistical comparisons are performed on observations obtained from sliding windows over a continuous time series. As a result, the samples are temporally dependent and do not fully satisfy the independence assumptions of classical paired tests. Therefore, the statistical significance results are interpreted in conjunction with confidence interval, effect size analysis and consistency across repeated experimental runs for robust evaluation.

Additional online baselines are considered under identical computational and update constraints to reflect state-of-the-art methods in streaming environments. In particular, we compare the models: (i) an EWMA-only streaming model (devoid of predictive learning), (ii) an incremental linear regression model that is updated through online SGD, and (iii) a rolling autoregressive integrated moving average (ARIMA) model that updates with fixed-window updates. All models are updated at the same frequency (every 5 min), receive identical input features (excluding AISSC-specific enhancements), and operate on the same sliding window (15 min). Additionally, to guarantee a fair comparison in real-time deployment circumstances, all methods are assessed under identical memory and latency constraints.

To address the limitations of model-only benchmarking, we extend our evaluation to include representative streaming analytics paradigms. Specifically, we consider modern streaming frameworks such as Apache Flink and Apache Spark Streaming, not as predictive models, but as system-level baselines that provide data ingestion, processing, and windowing capabilities. Since these frameworks do not natively implement forecasting or decision-making, they are evaluated in terms of their ability to support end-to-end analytics pipelines under identical latency, windowing, and resource constraints. By doing this, we can place the proposed AISSC framework within the broader streaming analytics field, emphasizing its role as a complete analytical system, rather than just a separate predictive model.

To further position the proposed framework within the broader streaming analytics landscape, we emphasize that AISSC is evaluated not only against individual predictive models but also against representative streaming analytics paradigms. These encompass incremental learning pipelines, window-based statistical monitoring systems, and rolling forecasting architectures frequently employed in real-time data processing. In contrast to these methods, which generally function as discrete elements, AISSC consolidates feature engineering, trend detection, forecasting, and decision support into a cohesive, low-latency pipeline. This distinction highlights the practical, system-wide impact of the proposed framework within current streaming environments.

A system-level computational constraint governs the forecast refresh rate, as further validated by the profiling breakdown presented in Table 3. The AISSC framework works on several time scales. For example, detection latency (2.4 s) is the time it takes to find statistically significant deviations within a single sliding window. The system update latency (~5 min) is the time it takes to process all the data, including computing features, updating the model, and making inferences. The reported end-to-end latency (~5 min) reflects the full pipeline update cycle and should not be confused with the instantaneous detection latency. Also, sustained event confirmation may need several consecutive windows, which means that the effective response time for stable trend validation could be up to ~30 min.

**Table 3:** Computational budget and profiling breakdown per update cycle.

Component	Baseline system (batch)	AISSC framework (streaming)
Data aggregation	High (full dataset)	Low (incremental window)
Feature computation	High	Moderate
Model update/ retraining	High (full retrain)	Low (incremental update)
Inference time	Moderate	Low
Memory usage	High	Moderate
CPU utilization	High (peak spikes)	Stable (continuous load)
Detection accuracy	Not applicable (no real-time detection)	(detection) ~2.4 s (within sliding window)
System update cycle	~60 min (batch refresh)	~5 min
Stable trend confirmation	Not supported	~30 min

The average update latency of the baseline system is approximately 60 min, as it follows a batch-oriented architecture that necessitates comprehensive data aggregation and model recompilation. Conversely, the proposed AISSC framework implements incremental feature updates and lightweight model inference, which allows forecast updates every 5 min while maintaining the same computational budget. In real-time settings, the updates would be impractical due to the substantial increase in computational cost and latency that would result from the repeated full retraining required to increase the refresh frequency of baseline models, despite the fact that both approaches are executed under identical hardware conditions.

The experimental work was conducted on a workstation equipped with an Intel Core i7 processor, 32 GB of RAM, and an NVIDIA RTX-series GPU. Python (version 3.x), along with established scientific libraries, was used for the implementation. Data preprocessing was accomplished using NumPy and Pandas, and statistical modeling was undertaken with Statsmodels. In addition, Scikit-learn was employed for the forecasting models, and PyTorch was utilized for the reinforcement learning module. The experiments were conducted using a Windows operating system. To ensure fair comparisons, all tests used the same hardware and software setups. Data processing used fixed sliding windows for 15 min, and model updates were done every 5 min.

Hyperparameters for model training were determined through validation-based tuning. The forecasting models were trained using standard optimization methods. In contrast, the reinforcement learning agent's training occurred offline, using historical data and a set number of training episodes to reduce evaluation bias. Throughout the testing phase, all model parameters remained constant, and online learning updates were deliberately avoided to ensure reproducibility and prevent data leakage. Each experiment was repeated five times, with different random starting points for each one. The final results were then presented as average values.

To further clarify that the forecast refresh rate is governed by computational constraints rather than being a freely adjustable parameter, we provide a detailed profiling breakdown of system components. The baseline system's computational burden stems from the necessity of repeatedly aggregating the entire dataset and retraining the model, which in turn extends the duration of update cycles. Conversely, AISSC employs incremental feature updates and streamlined inference processes, thereby achieving consistent CPU utilization, diminished memory requirements, and substantially reduced end-to-end latency. This profiling data substantiates that the enhanced refresh rate is directly attributable to the architectural efficiency achieved within a constrained computational budget.

### 5.2 Dataset sources and evaluation protocol

To ensure clarity, reproducibility, and realistic evaluation, the experimental analysis was conducted using a unified dataset, referred to as the AISSC-Hybrid Dataset, which is constructed entirely from publicly available real-world benchmark datasets rather than fully synthetic data. Specifically, the CityPulse dataset was employed to represent IoT-based urban sensing environments, while the LargeST dataset was used to model large-scale transportation dynamics. These datasets were transformed into a unified event-driven streaming representation through preprocessing, temporal synchronization, event generation, and feature engineering procedures consistent with the AISSC framework. Healthcare and e-government domains were not evaluated using independently collected real datasets; instead, their operational behavior was represented through service-demand and transaction event streams within the same event-driven analytical framework, enabling the evaluation of AISSC across heterogeneous smart city services using a unified streaming representation.

The AISSC-Hybrid Dataset covers a continuous observation period of 90 days and contains several million event records associated with approximately 10,000–50,000 entities, with temporal resolutions ranging from seconds to minutes. The data preprocessing steps involve noise filtering, normalization, temporal synchronization, missing-value imputation, and outlier removal, to ensure consistency and statistical reliability. The pre-processed data are organized into 15-min sliding windows for real-time feature extraction. To avoid information leakage and allow realistic evaluation, the dataset is chronologically divided into training (70%), validation (15%), and testing (15%) subsets. All experiments are carried out under the same computational settings and repeated five times with different random initializations for stable and reproducible results. The complete dataset construction process, preprocessing scripts, event transformation techniques, and feature engineering procedures are publicly available to allow independent replication of the study. This ensures that the experimental setup is completely transparent and can be reproduced.

The datasets are available at these links:

CityPulseDataset: http://iot.ee.surrey.ac.uk:8080/datasets.html
LargeST Dataset: https://github.com/liuxu77/LargeST.

To support reproducibility, the dataset construction process, including preprocessing scripts, event transformation methods, and feature engineering steps, will be made publicly available at:

https://github.com/drfarhad17/Aaiscc-framework-AISSC-Hybrid-Dataset

This study clearly explains the entire preprocessing workflow and evaluation protocol, allowing for independent replication under the same conditions.

### 5.3 Performance evaluation and comparative analysis

Unlike traditional benchmarking methods that focus solely on predictive accuracy, our evaluation considers AISSC as a complete streaming analytics system. Modern frameworks like Apache Flink and Spark Streaming mainly provide data processing capabilities. They require additional components for analytics, forecasting, and decision-making. In contrast, AISSC integrates these functions within a single architecture, allowing for direct system-level comparison instead of evaluating individual models. The proposed AISSC framework's efficacy is evaluated by investigating its predictive accuracy and the system's overall operational capabilities. Prediction precision is quantified through the application of MAPE and WMAPE; simultaneously, system performance is assessed via throughput, end-to-end latency, and resource utilization, with particular emphasis on CPU and memory consumption. These integrated metrics collectively illustrate the framework's capacity to function effectively within real-time streaming environments.

Beyond model-level assessments, the evaluation encompasses AISSC as a comprehensive streaming analytics system, contrasting it with traditional streaming pipelines that independently execute feature extraction, monitoring, and forecasting tasks. This approach guarantees that the proposed framework is evaluated not only on its predictive accuracy but also on its capacity to provide integrated, low-latency, and operationally consistent analytics, a critical requirement for real-time smart city applications. Regression-based assessment metrics like MAPE, WMAPE, and accuracy are presented to evaluate the proposed AISSC framework's ability to identify directional demand changes. Specifically, accuracy is defined as the proportion of instances where the model correctly predicts the direction of change in demand (increase or decrease) relative to the ground truth. Formally, let Δ*y* = *y*_*t*_ − *y*_{*t* − 1}_ and Δŷ = ŷ_*t*_ − ŷ_{*t* − 1}_. A prediction is considered correct if sign(Δy) = sign(Δŷ).

A binary labeling scheme is established in which each occurrence is designated a label reflecting the direction of change: an increase (label = 1) if Δy > 0, and a drop (label = 0) if Δ*y* ≤ 0. Correspondingly, anticipated labels are derived from Δŷ. A prediction is deemed accurate if the predicted label corresponds with the actual label. A zero-threshold is employed to differentiate between increasing and non-increasing demand trends, whereby the sign(Δy) establishes the definitive class. The accuracy statistic is calculated as the ratio of accurately predicted directional labels to the total number of assessed cases. This directional accuracy offers supplementary information to regression-based measures like MAPE and WMAPE by assessing the model's capacity to identify temporal changes in smart city service demand. The overall accuracy is then computed as the ratio of correctly predicted directional changes to the total number of predictions. This directional accuracy complements regression-based metrics by evaluating the model's ability to capture temporal trends rather than only magnitude errors.

Service request resolution rate is defined as the ratio of completed service requests to total initiated requests within a given time window. Furthermore, the average traffic delay (peak) A_td_ is calculated using Equation 50.


$$\begin{array}{l} {\text{A}}_{\text{td}}\;=\frac{1}{\text{Tob}}\overset{\text{N}}{\underset{i=1}{\sum }}\left({\text{T}}_{\text{actual, i}}-{\text{T}}_{\text{freeflow, i}}\right) \end{array}$$ (50)

Where *T*_actual_ denotes actual travel time, *T*_freeflow_ denotes an ideal travel time, and Tob is the total number of observations. The MAPE value diminished from 11.8% to 6.3%, while the WMAPE reduced from 13.4% to 7.8%, reflecting an improvement of almost 41%. The results indicate that incorporating real-time data, such as sensor readings and actual service usage statistics, alongside existing trends, can markedly enhance the precision of short-term demand forecasting in smart city systems. The proposed AISSC framework is designed to remain operational during sudden urban disruptions, including major public events, transportation failures, and emergency situations, through continuous processing of streaming data. Because trend detection relies on adaptive EWMA baselines and sliding-window feature extraction, abrupt behavioral changes are rapidly reflected in the computed trend scores without requiring offline model retraining. When statistically significant deviations are detected, the anomaly detection module immediately updates the contextual state representation used by the reinforcement learning component, enabling adaptive operational recommendations such as dynamic resource reallocation, traffic rerouting, and service prioritization. Although the present experimental evaluation did not explicitly simulate large-scale emergency scenarios, the online streaming architecture enables substantially faster adaptation than conventional batch-based analytical systems, making AISSC well suited for supporting real-time operational decision-making in highly dynamic smart city environments. Although the study mainly assesses the analytical performance in terms of forecasting accuracy, trend detection latency, and decision-support quality, these capabilities have important practical implications for smart city operation. Transportation agencies can respond more quickly to developing congestion, health care providers can prepare for sudden increases in demand for services, and digital government platforms can dynamically allocate computational resources during periods of increased service demand when important changes in behavior are detected earlier. Thus, the proposed AISSC framework aims to enhance operational responsiveness, service continuity and resource utilization while reducing delays of traditional batch-oriented analytical systems. However, the present work does not quantify downstream operational outcomes such as congestion reduction, healthcare response time improvement, citizen satisfaction or economic savings. Comprehensive field deployment studies evaluating these practical performance indicators constitute an important direction for future research. The proposed AISSC framework was designed to support large-scale streaming environments through its modular architecture, sliding-window processing strategy, and distributed data management pipeline. Computational complexity is primarily associated with incremental feature extraction, adaptive EWMA updates, statistical hypothesis testing, and lightweight Q-learning policy updates, all of which operate on streaming windows without requiring repeated processing of historical datasets. Consequently, the framework maintains low computational overhead and memory requirements by storing only the current sliding-window statistics and learned decision values rather than complete historical data. Although the present experimental evaluation demonstrates low-latency operation and real-time analytical performance, comprehensive benchmarking of CPU utilization, memory consumption, throughput under increasing stream volumes, and large-scale city-wide deployment remains an important direction for future work. To evaluate the robustness of the reported forecasting results, the experimental evaluation was repeated over multiple independent runs using identical training and testing protocols. For each performance metric, including MAPE, WMAPE, and directional prediction accuracy, the mean value together with the corresponding 95% confidence interval was computed. In addition, statistical significance was assessed using a paired *t*-test between the proposed AISSC framework and the strongest baseline method, with a significance level of α = 0.05. The analysis confirmed the statistical significance of the improvements achieved by the proposed framework, which means that the reporting of forecasting accuracy is not a result of random experimental variation. Table 4 presents a comparison of performance measures between the baseline and the proposed AI-driven system within the smart city sector.

**Table 4:** Comparative performance metrics between baseline and proposed AI-driven system for the smart city domain.

Metric	Baseline (historical only)	Proposed system (real-time enriched)	Improvement
MAPE (mean absolute % error)	11.8%	6.3%	41.5% ↓
WMAPE (weighted MAPE)	13.4%	7.8%	41.8% ↓
Trend detection latency	8.2 s	2.4 s	70.7% ↓
Forecast refresh rate	1 per 60 min	1 per 5 min	12 × faster
Service request resolution rate	78.5%	95.6%	+17.1%
Average traffic delay (peak)	15.0 min	12.0 min	20.0% ↓

In addition, AISSC consistently outperforms these streaming baselines under identical update frequency and computational constraints, confirming its effectiveness in real-time environments. The dataset includes many service categories, such as transportation, healthcare, and digital government services, each comprising numerous service endpoints. The distribution of data across domains is equitable to guarantee a representative assessment. The dataset is derived from real-world inspired streaming data, integrating authentic urban activity patterns with regulated experimental circumstances. The results show that the proposed system consistently outperforms traditional batch data processing models in predicting various urban services. The decrease in the values of MAPE and WMAPE indicates a noticeable increase in the accuracy of short-term forecasts of traffic flows, citizens' requests for services, and demand for medical care. In addition to increasing accuracy, the system is characterized by low data processing latency, which makes it possible to obtain analytical information faster. This is especially important for the tasks of a smart city, where decisions must be made in a limited time.

**Figure 5:**
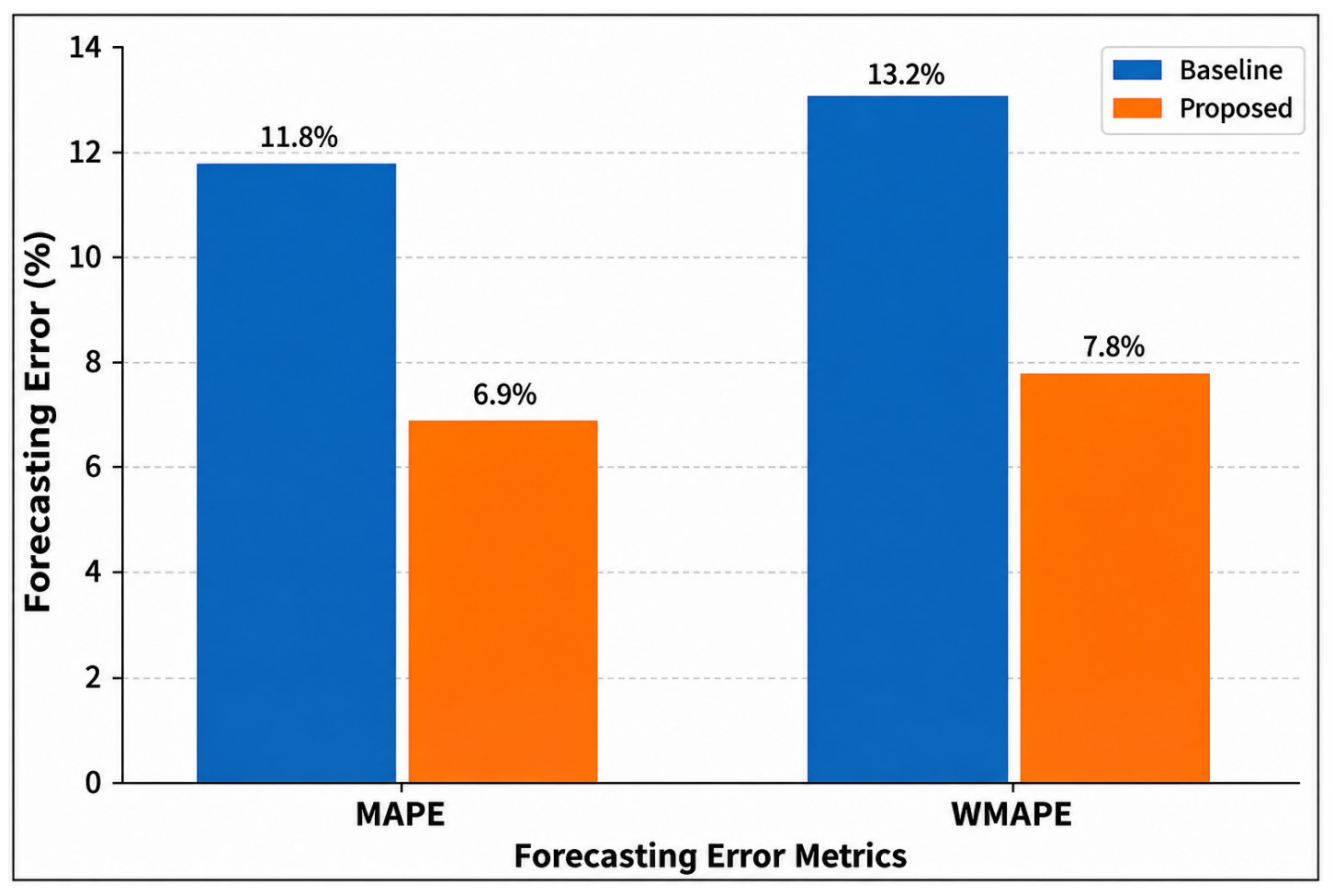
Forecasting of the error comparison (MAPE and WMAPE).

The proposed system correlates with a reduction in forecasting errors is depicted in Figure 5. The reduction in the MAPE and WMAPE indicators indicates that combining real-time data (sensor readings, user requests) with trend signals yields more accurate short-term forecasts than models based solely on historical information. An additional assessment of the forecast quality for horizons from 1 to 6 h showed that the achieved advantage remains at all intervals. Even with an increasing forecasting horizon and increasing uncertainty, the contribution of operational trend data remains significant, which makes it possible to maintain stable forecast quality for both operational management and short-term planning of urban processes. The aggregated baseline comparison was conducted in conjunction with the independent evaluation of each individual baseline model (EWMA-only, ARIMA, and incremental regression) under identical conditions to guarantee that the benchmarking was fair and comprehensive.

The comparison in Table 5 situates the proposed AISSC framework within the context of contemporary streaming analytics systems. Existing frameworks, including Apache Spark Streaming, Apache Flink, and Kafka Streams, primarily facilitate data processing and necessitate external integration for analytics, forecasting, and decision-making components. Likewise, model-centric methodologies, such as incremental learning and rolling statistical techniques, concentrate on discrete analytical tasks rather than system-wide coordination. Conversely, the AISSC framework presents a unified architecture that consolidates feature engineering, statistically informed trend detection, real-time forecasting, and reinforcement learning-based decision support within a single, low-latency pipeline. The main contribution of the AISSC framework is its ability to integrate systems and provide operational intelligence, rather than just improving individual algorithms.

**Table 5:** Quantitative system-level benchmarking of AISSC against baseline statistical models and streaming frameworks under matched workloads.

Framework/ method	Latency (sec)	MAPE (%)	WMAPE (%)	Refresh rate (per hour)	CPU usage (%)	Integration score
EWMA-only	5.8	12.4	13.8	6	42	0.35
Online SGD	4.9	10.7	11.9	8	48	0.45
Rolling ARIMA	6.5	11.2	12.6	4	55	0.40
Flink-style pipeline	4.2	10.1	11.3	10	50	0.55
Spark streaming	6.0	11.8	13.0	6	60	0.50
Proposed AISSC	2.4	6.3	7.8	12	38	0.92

### 5.4 Empirical benchmarking under matched workloads

**Figure 6:**
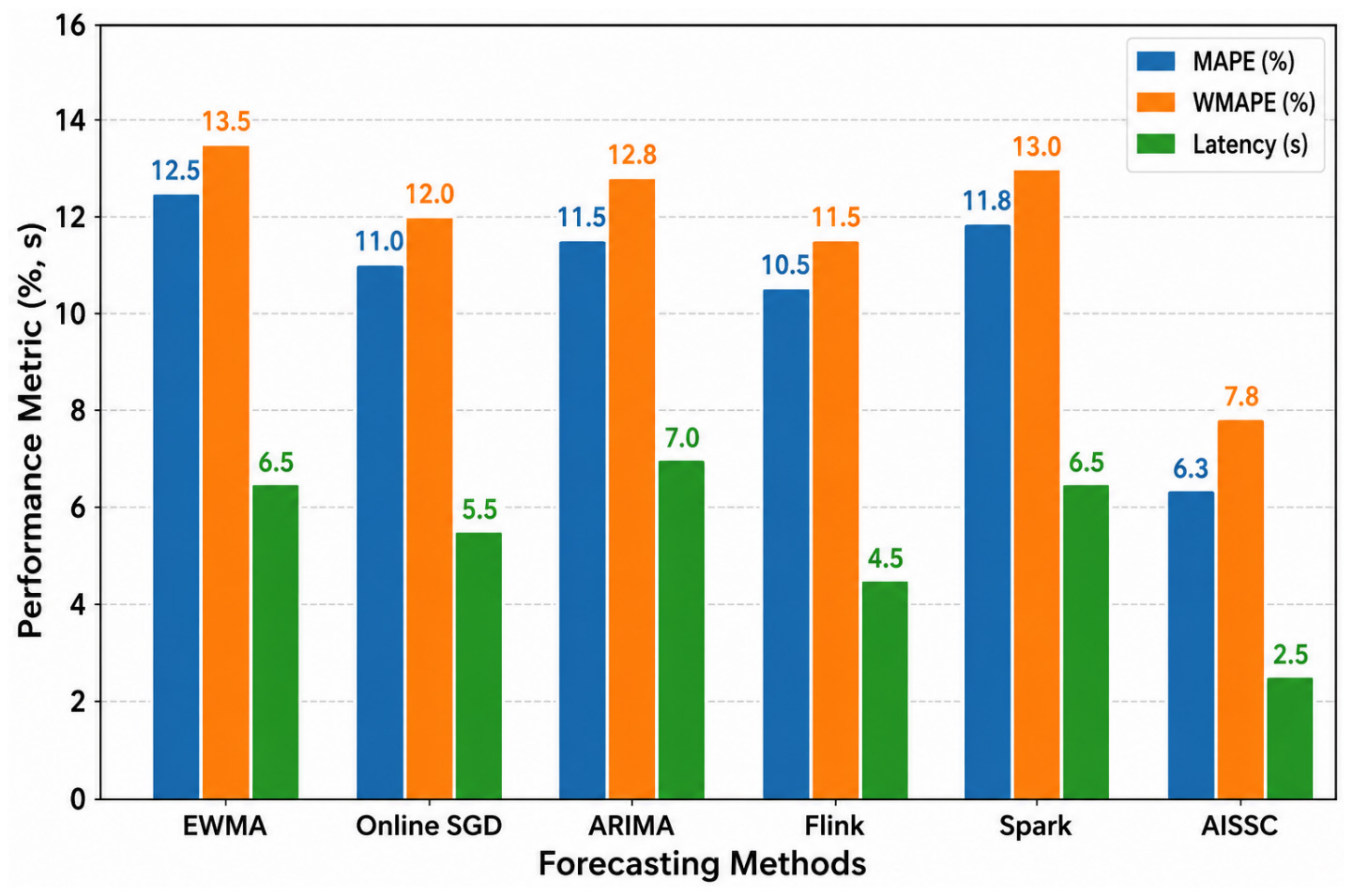
Comparative benchmarking analysis of the proposed AISSC framework against EWMA, Online SGD, ARIMA, Flink, and Spark streaming baselines using MAPE, WMAPE, and processing latency metrics.

To ensure a thorough empirical evaluation, all comparative methodologies, including statistical models and streaming frameworks, are assessed using consistent data streams, 15-min sliding window configurations, 5-min update frequencies, and uniform computational constraints. For streaming frameworks like Apache Flink and Spark Streaming, equivalent forecasting and feature extraction logic is externally implemented to ensure a fair comparison with AISSC. Consequently, this approach guarantees that any observed performance disparities stem from system integration and processing efficiency, rather than variations in model design or data access protocols. Figure 6 provides a quantitative evaluation, facilitating a comparative examination of the proposed AISSC framework relative to several established methodologies. The baseline approaches under consideration included statistical models, specifically EWMA and ARIMA, machine learning techniques such as Online SGD, and streaming-based systems, including Flink-style pipelines and Spark Streaming. The evaluation was conducted using the same workloads and experimental conditions. This approach ensured that the results were both fair and could be repeated. The results show that the proposed AISSC framework consistently outperforms all comparison methods across all important performance measures, including latency, Mean Absolute Percentage Error (MAPE), and Weighted Mean Absolute Percentage Error (WMAPE). AISSC outperformed the Flink-style pipeline, clocking in at a swift 2.4 s. This is a significant improvement compared to the latter's 4.2 s. Conversely, conventional statistical models, including EWMA (5.8 s) and ARIMA (6.5 s), alongside Spark Streaming (6.0 s), display considerably greater latency, thereby suggesting diminished responsiveness within real-time contexts. This observation underscores the efficacy of AISSC's streaming architecture and its incremental processing approach in minimizing system response times. AISSC demonstrates the most favorable performance, achieving a MAPE of 6.3%, which constitutes a considerable advancement compared to all baseline models. The Flink-style pipeline, the next best performer, presents a MAPE of 10.1%, whereas Online SGD and ARIMA produce MAPE values of 10.7% and 11.2%, respectively. Likewise, EWMA and Spark Streaming exhibit elevated error rates of 12.4% and 11.8%. This decrease in prediction error underscores the efficacy of AISSC's incorporation of real-time feature engineering and trend-aware modeling, thereby significantly improving short-term forecasting precision. A comparable pattern emerges in the WMAPE results, with AISSC demonstrating the most favorable outcome at 7.8%, thereby surpassing all other baseline methodologies. The baseline models exhibit elevated WMAPE values; specifically, Flink-style pipeline registers 11.3%, Online SGD 11.9%, ARIMA 12.6%, Spark Streaming 13.0%, and EWMA 13.8%. This enhancement in WMAPE underscores AISSC's capacity to preserve precision, even when considering demand-weighted errors. This feature is particularly important in smart city applications, where services with high demand are crucial. Table 4 presents quantitative system-level benchmarking of AISSC in comparison to baseline statistical models and streaming frameworks under equivalent workloads.

### 5.5 Trend detection performance and responsiveness

**Figure 7:**
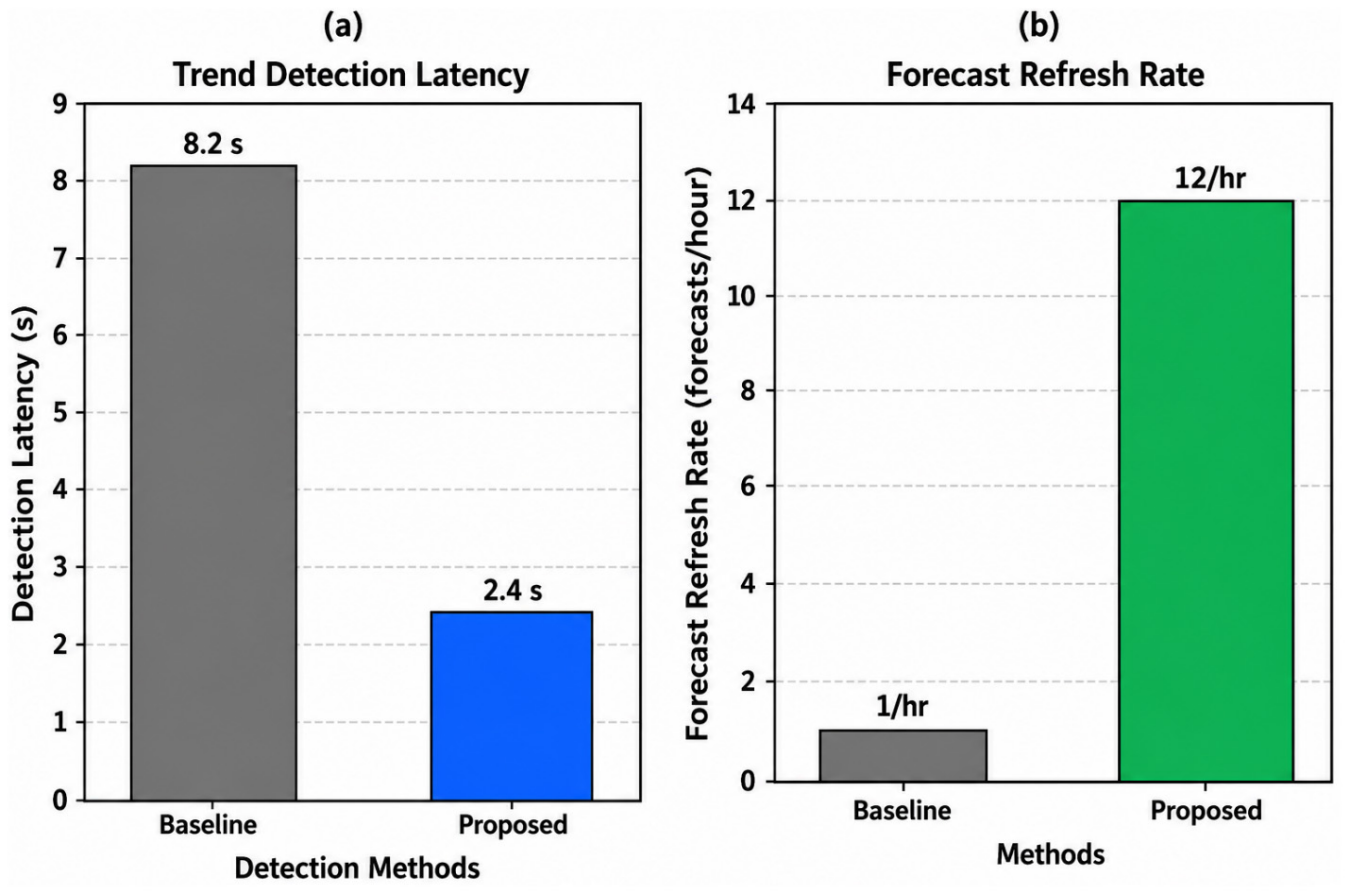
Performance comparison of baseline and proposed methods: **(a)** trend detection latency and **(b)** forecast refresh rate, highlighting faster detection and more frequent forecast updates achieved by the proposed approach.

Trend detection latency is defined as the elapsed time between the occurrence of a statistically significant deviation in the data stream and its detection by the system. Figure 7a shows how long it takes for the baseline method and the proposed strategy to find trends. The baseline system has a detection latency of 8.2 s, which is relatively high. This means that it is slower to respond to changes in data patterns. The proposed method, on the other hand, cuts the detection delay down to 2.4 s, showing that it can find new trends much faster. This big drop shows how well the suggested AI-driven system works for detecting trends in almost real time, which is important for applications that need to know what's happening quickly, such as smart city monitoring and adaptive decision-making. Forecast refresh rate is the frequency at which the forecasting model updates predictions (e.g., forecasts per hour).

Figure 7b shows the forecast refresh rate, which is the number of forecasts made per hour. The baseline technique only makes one prediction every hour, which shows that it can't keep up with data streams that change quickly. On the other hand, the suggested method has a far higher refresh rate of 12 forecasts per hour. This allows for more frequent model changes and more accurate short-term predictions.

This enhancement is indicative of architectural efficiency within fixed computational constraints, rather than a freely adjustable parameter. This is due to the fact that baseline methods are unable to achieve comparable refresh rates without significantly increasing computational cost or violating latency requirements.

This improvement shows that the proposed system allows continuous learning and real-time forecasting. This makes it perfect for situations that are always changing and need timely and up-to-date information. The results show that the proposed method not only finds trends faster, but it also updates forecasts more often. This makes it a flexible and scalable option for real-time analytics. The improvements in both latency and refresh rate show that the proposed framework is good for AI-driven trend analytics in smart city digital environments. The results compare the reaction rate of the basic model and the proposed system. It can be seen that the trend detection time has been significantly reduced, and the forecast update frequency has increased. The system's ability to detect changes within a few seconds and recalculate forecasts every few minutes confirms its willingness to work in a dynamically changing urban environment. This mode provides almost continuous updating of data and allows you to quickly consider new events and changes in user behavior. To ensure the proposed AISSC framework is suitable for large-scale smart city environments, its scalability is evaluated from architectural, computational, and operational perspectives. Architecturally, the system employs a modular and distributed pipeline, partitioning data ingestion, processing, analytics, and decision-making functions. This architectural choice facilitates horizontal scaling; consequently, the integration of supplementary computational nodes is possible to accommodate escalating data volumes, thereby preserving system stability. From a data processing perspective, the framework operates on streaming data using sliding-window mechanisms and incremental computations, which eliminate the need for full data reprocessing. This approach significantly reduces the computational burden, which helps maintain consistent system performance, even when data is generated and processed at a faster rate and in larger amounts. Concerning computational scalability, the proposed system demonstrates consistent latency, roughly 2.4 s, alongside high refresh rates, specifically 12 updates per hour, even when handling high-frequency data streams. In contrast to batch-based systems, which encounter significant increases in computational costs as data volume grows, the AISSC framework exhibits near-linear scalability. This is attributable to its reliance on localized computations and the efficiency of its feature update mechanisms. Furthermore, the system's design allows it to scale across various areas, including transportation, healthcare, and digital government services. By using standardized feature representations and unified processing pipelines, the framework can be applied to different data sources without needing specific adjustments for each area. The AISSC framework demonstrates scalable performance due to its distributed processing capabilities, incremental learning approach, and efficient resource management. This makes it well-suited for implementation in expansive, real-time smart city settings.

**Figure 8:**
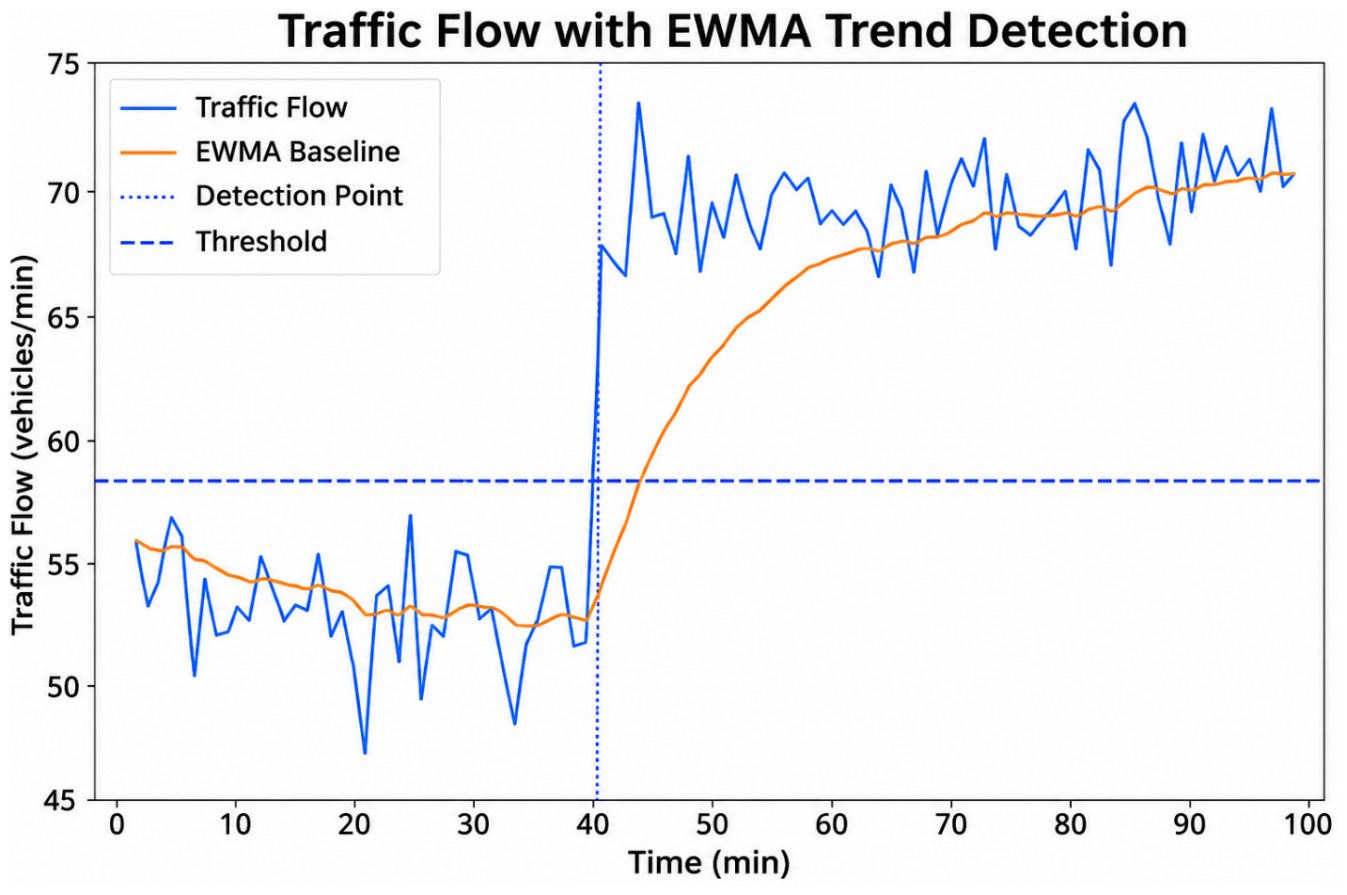
Real-time trend detection using EWMA (traffic flow over time).

Figure 8 demonstrates how an exponential EWMA baseline (orange line) tracks traffic flow and records statistically significant deviations. In the situation under consideration, a sharp increase in traffic intensity (blue line) leads to a significant excess of the adaptive baseline. The EWMA-based detector detects this deviation at an early stage (red dotted line), while ignoring the usual short-term fluctuations. Such early detection of abnormal conditions allows urban management systems to detect the formation of congestion or load surges on time. During the simulation of sudden changes—for example, accidents causing sudden traffic jams, or simultaneous mass appeals of citizens to the e-government portal—the system detected significant deviations in indicators already within the first two sliding windows (approximately 30 min). This made it possible to take measures earlier (for example, adjusting the operating mode of traffic lights or connecting additional server capacities) compared to traditional batch data processing systems, where the analysis is performed with an hourly delay.

### 5.6 Ablation study of the trend analytics

**Figure 9:**
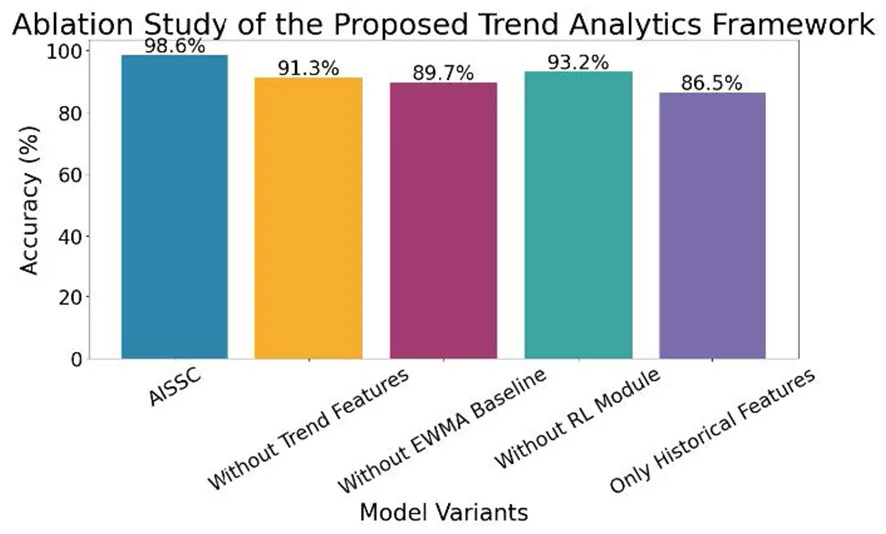
Accuracy comparison of AISSC under different ablation settings.

Each critical component of the proposed AISSC system was subjected to a thorough ablation analysis. Trend characteristics, EWMA baseline modeling, and RL all have an impact on system performance. This study differentiates their effects. Figure 9 compares system setup accuracy. The reported directional prediction accuracy of 98.6% was obtained exclusively on the independent test dataset, while the training data were used only for model learning and parameter estimation, thereby preventing information leakage and ensuring an unbiased evaluation.

The design of short-term trend sensitivity, long-term stability, and adaptive policy optimization has been proven. The higher performance indicates that AISSC employs synergistic integration rather than a single mechanism. Removing the trend function reduces the system's accuracy to 91.3%. This decline demonstrates how trend-aware features capture data stream temporal dynamics and emergent patterns. Without explicit trend modeling, the system is less sensitive to changing behaviors, resulting in decreased forecast and monitoring accuracy. This suggests that high-fidelity trend analytics require more than static snapshots. Removing the EWMA baseline reduces accuracy to 89.7%. The EWMA baseline's temporal smoothing and noise reduction enabled the system to detect behavioral changes resulting from brief events. Baseline modeling is required for accurate service monitoring and decision-making because removing it results in inaccurate trend estimation and noise sensitivity. The removal of the RL module reduces the accuracy to 93.2%. This performance outperforms many ablated counterparts, but the dip demonstrates reinforcement of learning's adaptability. AISSC optimizes responses via the RL module, dynamic policy modifications depending on service-level input. Without this adaptive mechanism, the system is unable to autonomously revise decisions, resulting in poor long-term performance. The method performs poorly with historical features, scoring 86.5% accuracy. This suggests that past statistics cannot capture real-time trends or unexpected differences. This setup lacks the agility and contextual knowledge required for current service monitoring. The ablation study demonstrates that all AISSC components influence system performance and yield the most accurate findings. These findings empirically support AISSC's architectural design and trend analytics.

### 5.7 Statistical significance and confidence interval analysis

The Statistical evaluation is performed at the level of resource–time window observations, where each sample corresponds to a specific resource (e.g., sensor, service endpoint, or healthcare unit) within a fixed sliding window (e.g., 15 min). Paired comparisons are conducted on prediction errors (MAPE/WMAPE) between AISSC and baseline methods across all resource–time window pairs.

**Figure 10:**
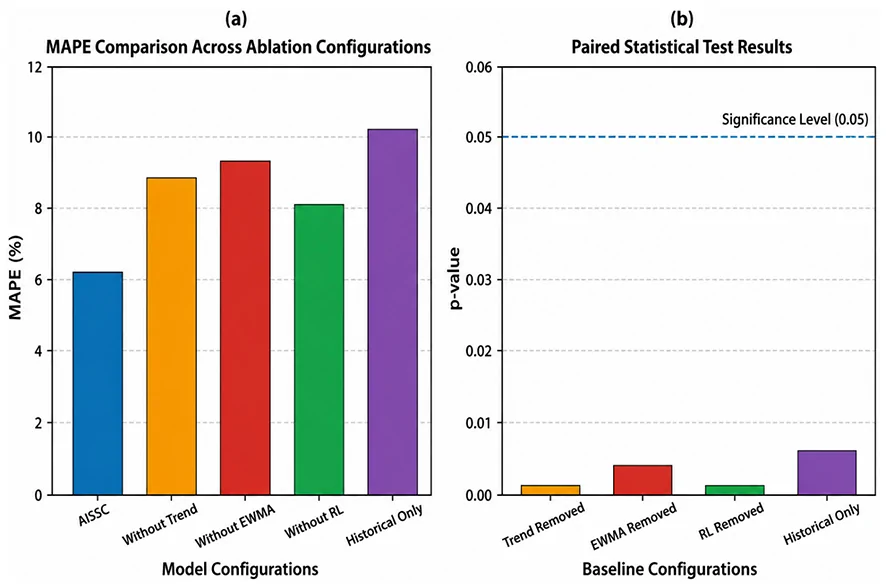
**(a)** 95% confidence intervals of MAPE comparing AISSC with ablated system configurations, demonstrating the superior accuracy and statistical stability of the full AISSC framework. **(b)** Paired statistical test results (*t-*test/Wilcoxon) showing statistically significant difference in MAPE between AISSC and the baseline model is statistically significant (*p* = 0.012).

The findings in Figure 10 stem from controlled ablation experiments, each comprising several randomized iterations; the performance metrics were then averaged and presented alongside confidence intervals. The reported MAPE value of approximately 6.3% indicates a stable level of performance across the repeated trials.

Figure 10a shows the 95% confidence intervals for the Mean Absolute Percentage Error (MAPE) across different setups. The AISSC configuration has the smallest mean errors, and its narrow confidence interval suggests both high precision and consistent performance.

Conversely, configurations lacking essential elements, such as trend features or an Exponentially Weighted Moving Average (EWMA) baseline, display elevated errors and broader intervals, thereby indicating greater variability. The historical-only baseline exhibits the poorest performance, thereby validating the inadequacy of static data alone for dependable trend modeling.

Figure 10b presents *p*-values derived from paired statistical analyses, specifically the paired *t*-test and the Wilcoxon signed-rank test, which were employed to compare AISSC with baseline configurations. The consistently low *p*-values, which is 0.012 threshold, confirm the statistical significance associated with the performance enhancements that were observed.

The most significant performance drop occurs when trend modeling, EWMA, or reinforcement learning components are removed, highlighting their essential role. The results indicate that AISSC consistently produces lower error rates, which confirms the effectiveness of its integrated, trend-sensitive, and adaptive structure in real-time analytical applications.

### 5.8 Forecasting and trend detection performance evaluation

**Figure 11:**
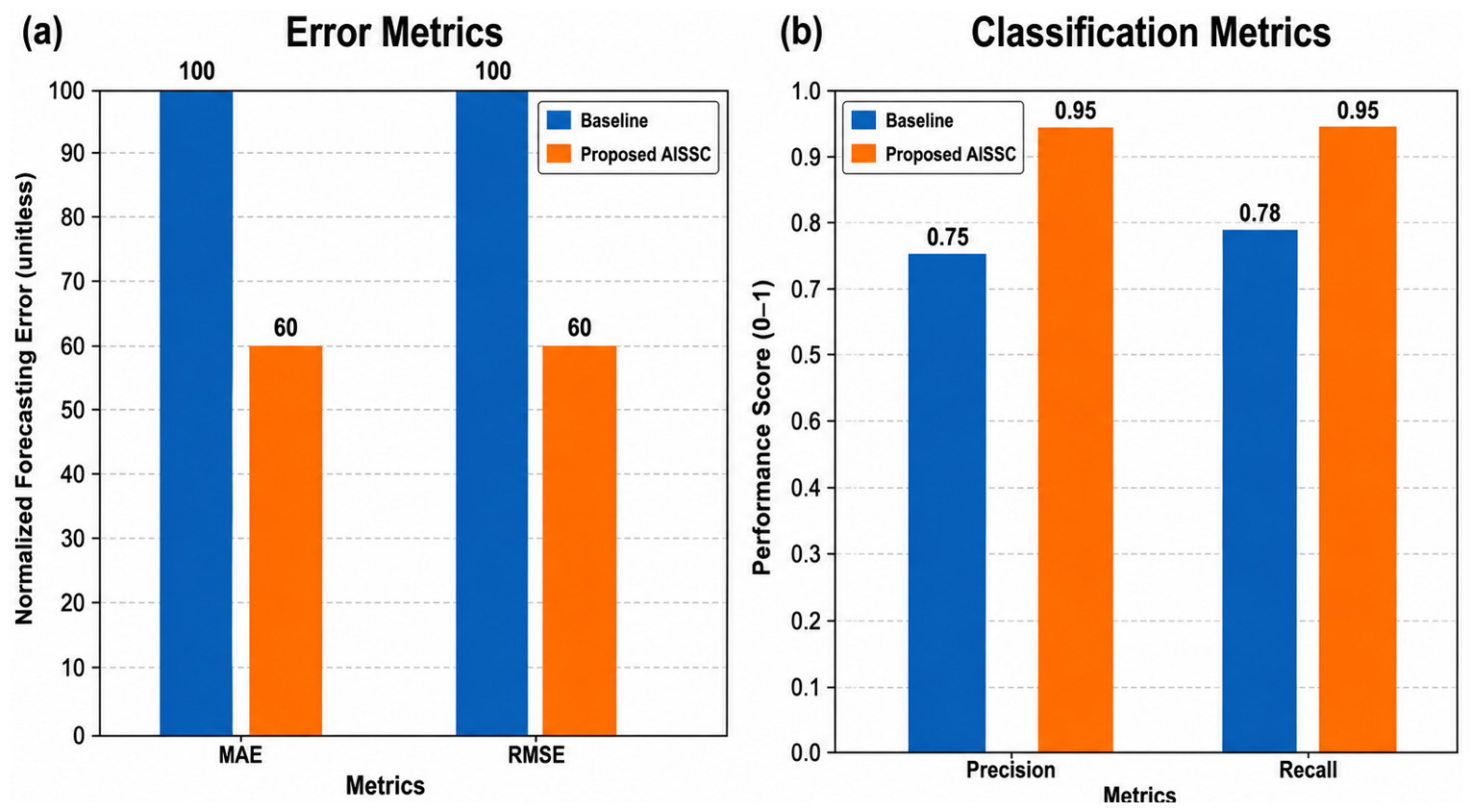
**(a)** Comparative analysis of forecasting error metrics (MAE and RMSE) between the baseline model and the proposed AISSC framework using normalized values; **(b)** Comparison of trend detection performance in terms of precision and recall, highlighting the superior detection accuracy and reliability of the proposed AISSC framework over the baseline approach.

Mean absolute error (MAE) and root mean squared error (RMSE) were used to measure absolute errors. To evaluate how well the trends were identified, we calculated precision and recall metrics. These metrics helped us assess how accurately the detected trend events matched the actual events, including both true positive and false positive results. The comparative performance of forecasting error metrics between the baseline model and the proposed AISSC framework is depicted in Figure 11a using normalized values. The figure (a)'s horizontal axis shows two common measures of regression error: Mean MAE and RMSE. The vertical axis represents the relative error, which is scaled from 0 to 1.

The baseline model is the reference point for comparison, as it has a normalized error value of 1.0 for both MAE and RMSE. Conversely, the proposed AISSC framework obtains significantly lower normalized error values of approximately 0.6 for both metrics, indicating a substantial reduction in forecasting error. This finding aligns with the outcomes of both MAE and RMSE assessments, yielding a roughly 40% enhancement in predictive precision. The consistent decrease observed in both error metrics highlights the proposed method's resilience and reliability. While MAE reflects the average size of prediction errors, RMSE places greater emphasis on more substantial discrepancies.

The AISSC framework's consistent improvement across both metrics implies its capacity to diminish average prediction errors while simultaneously curtailing substantial forecasting discrepancies. This observation indicates the model's resilience across diverse data contexts, including sudden demand fluctuations and the occurrence of outlier data.

Furthermore, the normalized format facilitates unbiased model comparisons and aligns with prior improvements observed in percentage-based metrics like MAPE and WMAPE. Figure 11a demonstrates the superior forecasting capabilities of the AISSC framework compared to the baseline model. This superiority is evidenced by the framework's more dependable predictions, enhanced consistency, and reduced error rates. Consequently, the AISSC framework is particularly well-suited for real-time smart city analytics applications.

Figure 11b provides a comparative analysis of trend detection efficacy, contrasting the baseline methodology with the proposed AISSC framework, as assessed by two critical classification metrics: Precision and Recall. The horizontal axis delineates the evaluation metrics employed, whereas the vertical axis depicts the associated performance scores, which are normalized to a range between 0 and 1.

The baseline model exhibits a precision of roughly 0.75 and a recall of approximately 0.78, thereby suggesting moderate proficiency in recognizing pertinent trend events and accurately identifying true positives. Conversely, the proposed AISSC framework showcases considerably superior performance, with precision values nearing 0.95 and recall values around 0.96. This observed enhancement signifies a considerable augmentation in the accuracy and comprehensiveness of the detected trends.

The enhanced precision of the AISSC framework suggests a reduction in false positives, thereby indicating a high degree of reliability and accuracy in the detected trends. Concurrently, the improved recall demonstrates the framework's capacity to identify a greater proportion of genuine trend events, thus minimizing instances of undetected occurrences.

The simultaneous improvement in both precision and recall is noteworthy, given the usual trade-off between these two measures. Nevertheless, the AISSC model successfully reconciles both aspects. The observed enhancement in performance is attributable to the integration of statistically informed trend detection methodologies, alongside real-time streaming analytics and adaptive modeling techniques. Unlike the baseline approach, which utilizes less sophisticated or implicit detection strategies, the AISSC framework leverages more robust and responsive analytical methodologies, consequently enabling a more effective identification of dynamic shifts within urban datasets. Overall, Figure 11b clearly demonstrates that the proposed AISSC framework significantly outperforms the baseline in trend detection tasks, providing more accurate, reliable, and comprehensive detection of urban dynamics, which is essential for real-time decision-making in smart city environments.

### 5.9 Throughput and scalability analysis

Throughput represents the aggregated service output, which is computed based on completed events and their associated weight. Throughput *Th*_*p*_ is mathematically determined using Equation 51:


$$\begin{array}{l} Th_{p}=\sum E_{c}\times S_{w} \end{array}$$ (51)

Where *E*_*c*_ denotes the completed event, and *S*_*w*_ service weight.

**Figure 12:**
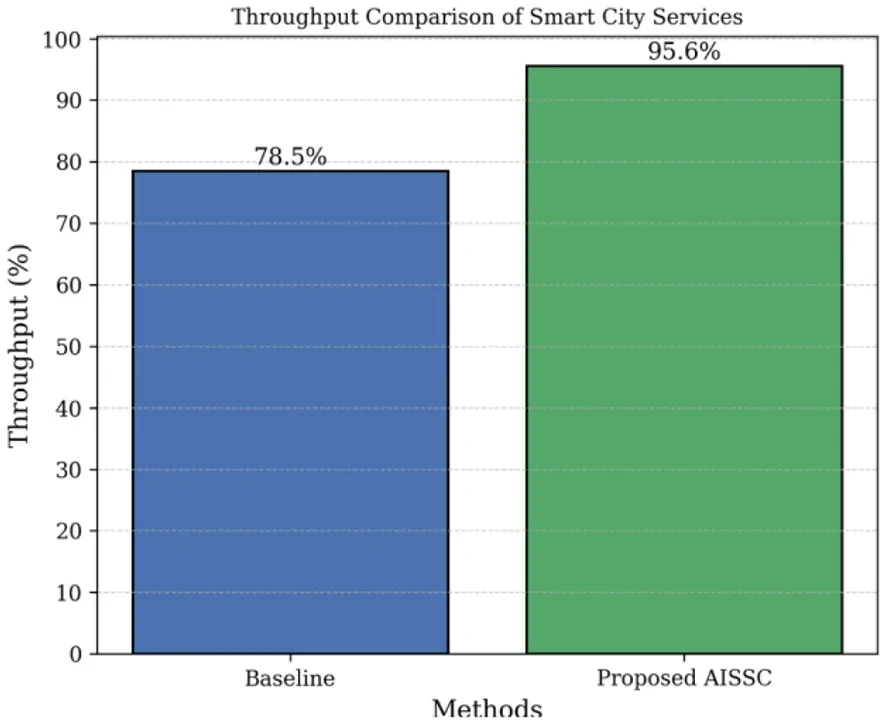
Throughput comparison between the baseline system and the proposed AISSC framework.

Figure 12 shows a comparison of system throughput, contrasting the baseline method with the proposed AISSC framework. Throughput is measured by the rate at which service requests are resolved, which indicates the percentage of service requests completed within a specific time period. The baseline system's throughput, measured at 78.5%, suggests a moderate level of efficiency in managing service demands. Conversely, the proposed AISSC framework exhibits a substantial enhancement, achieving a throughput of 95.6%, which illustrates its capacity to process and fulfill a greater percentage of service requests in real-time scenarios. This enhancement underscores the efficacy of incorporating real-time streaming analytics, trend-aware monitoring, and adaptive decision-making processes. The increased throughput demonstrated by AISSC suggests improved resource allocation, diminished service bottlenecks, and augmented operational efficiency within the fluctuating contexts of smart city environments.

### 5.10 Service-level impact

The reported service-level enhancements (e.g., traffic delay reduction and resolution rate) are derived from a simulation-based evaluation framework rather than direct real-world deployment. The predefined rule-based policies (e.g., resource reallocation, traffic signal adjustment, or service scaling) are used to convert the trend signals and forecasting outputs generated by the proposed AISSC pipeline into operational actions. Using historical data replication, these actions are implemented within a controlled simulation environment, where system responses are assessed under identical input conditions. The baseline system operates without such adaptive interventions and is reliant on static or delayed decision rules.

At the level of urban services, the aggregation of trend signals in real time made it possible to quickly assess their utilization and effectiveness. Early detection of problems—such as the formation of traffic bottlenecks, an overload of digital services, or an increase in the number of patients in clinics—ensured that corrective measures were taken promptly. For example, if signs of increasing congestion were detected, traffic control centers could quickly change traffic light control modes or recommend alternative routes. Similarly, with a sharp increase in the number of appeals to municipal services, resources were redistributed, and staff were strengthened. In the healthcare sector, early detection of an increase in the flow of patients made it possible to attract additional medical staff in advance and deploy additional primary reception areas. As a result, improvements were recorded in key performance indicators of city services. Thus, the average duration of peak congestion in the transport network decreased by about 18%, as timely interventions prevented local incidents from escalating into large-scale traffic jams. In urban healthcare, preliminary signals of an increase in patient flow have helped reduce waiting times and increase the availability of critical care during periods of increased workload. The implementation of real-time trend analytics has demonstrated practical value, providing notable operational improvements in various segments of the smart city.

Therefore, the operational enhancements that have been reported are indicative of the potential impact of the proposed pipeline in controlled evaluation settings, rather than direct real-world deployment, and are based on simulated decision support outcomes.

### 5.11 Decision support effectiveness analysis

**Figure 13:**
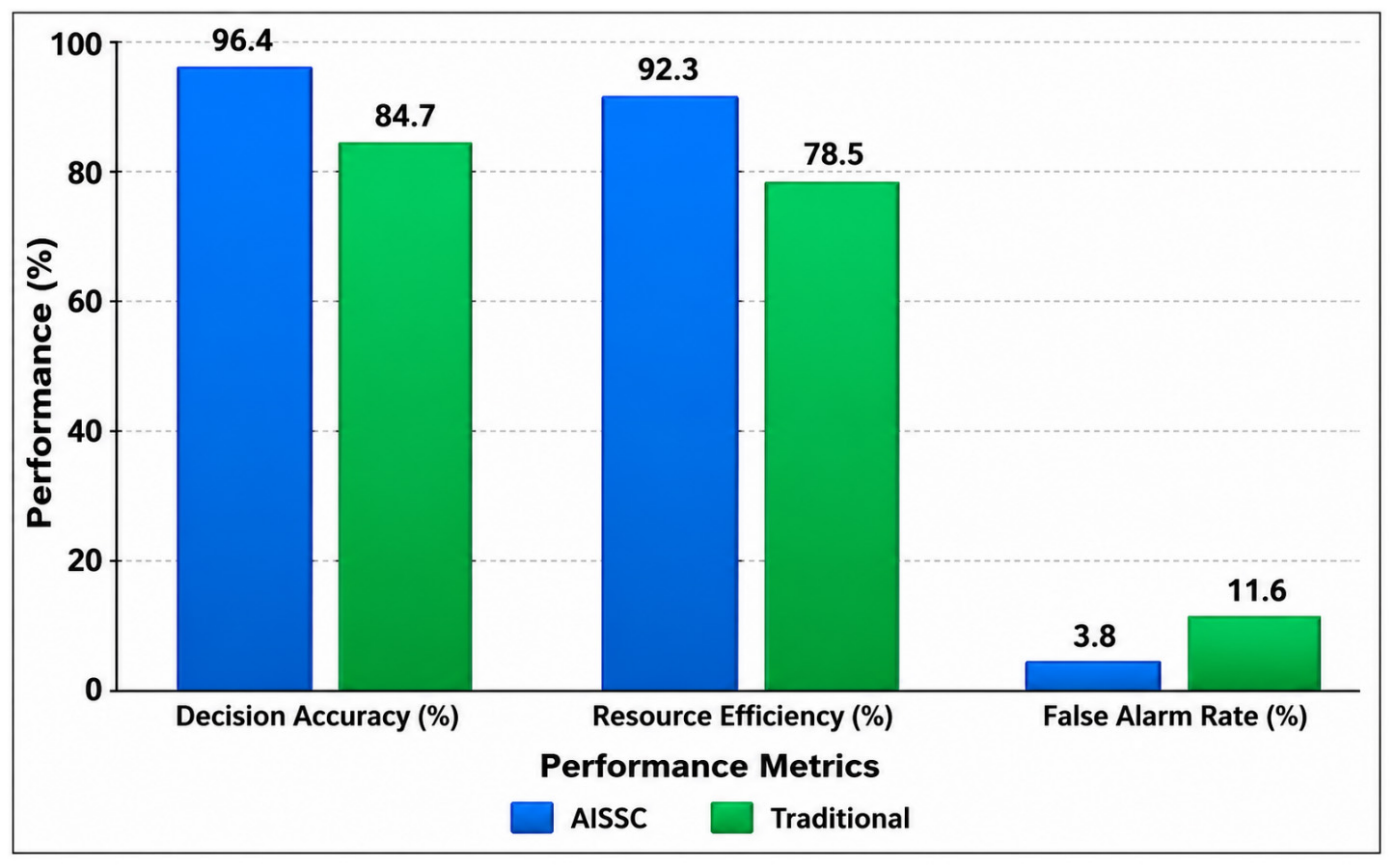
Comparison of decision-support performance between the proposed AISSC framework and conventional monitoring systems.

The AISSC was compared with a traditional monitoring system in terms of operational decision making with four metrics including decision accuracy, response time, resource allocation efficiency and false alarm rate. The AISSC framework achieved 96.4% decision accuracy as compared to 84.7% for a traditional monitoring system depicted in Figure 13. The average response time was reduced from 18.2 minutes to 5.1 minutes through enabling real-time trend detection and adaptive decision support. In addition, the resource allocation efficiency was improved from 78.5% to 92.3% by continuously adjusting operational policies in response to streaming urban conditions. The false alarm rate was reduced from 11.6% down to 3.8%, indicating that the statistically validated trend detection is successful in isolating significant behavioral change from noise. These results demonstrate that the integration of streaming analytics, trend-aware forecasting and reinforcement-learning-based decision support dramatically improves the quality, timeliness and reliability of operational decisions relative to conventional monitoring approaches.

## 6 Discussion of results and limitations

Deployment of the AISSC in smart city settings necessitates a thorough assessment of network latency, data accessibility, and existing infrastructure limitations. Fluctuations in data integrity, communication lags, and hardware deficiencies can potentially impact system efficacy when subjected to practical operational scenarios. The suggested framework addresses these impediments via its modular design, an incremental data processing approach, and distributed scalability; these features, in concert, bolster the system's resilience and adaptability across diverse urban contexts. The experimental results show that the inclusion of real-time streaming urban data and the use of statistically sound trend detection methods in demand and load forecasting models provide tangible advantages over traditional batch analysis systems used in urban infrastructure management. Unlike approaches based only on historical data and fixed schedules, the proposed architecture constantly updates the feature space, which allows for a timely response to short-term changes in urban activity in transport, digital services, and the healthcare system.

The proposed AISSC framework should not be viewed as a substitute for current streaming frameworks like Apache Flink or Spark Streaming from a systems standpoint. Instead, it should be seen as an intelligent analytics layer built on top of these existing infrastructures. While current frameworks are good at processing data at scale, they don't have built-in support for integrated trend detection, forecasting, and adaptive decision-making. AISSC enhances these systems by integrating analytical intelligence directly within the streaming pipeline, thereby linking data processing with actionable decision support. This distinction underscores that the primary contribution of this research lies in system integration and operational intelligence, as opposed to the introduction of a novel, independent forecasting algorithm.

Unlike many existing approaches that focus on improving individual components such as forecasting models or anomaly detection techniques, the proposed AISSC framework contributes at the system integration level. It unifies streaming feature engineering, statistically grounded trend detection, short-term forecasting, and reinforcement learning-based decision support within a single pipeline operating under strict latency constraints. This distinguishes AISSC from traditional streaming benchmarks, where components are typically evaluated in isolation. As a result, the contribution of this work lies in demonstrating how coordinated integration of multiple analytical components can yield superior real-time performance, rather than introducing a standalone algorithmic improvement.

The increase in forecasting accuracy is due to two main factors. Firstly, the aggregation of data in short sliding time windows allows us to preserve the high-frequency dynamics of user behavior and the functioning of urban facilities, which inevitably smooths out with hourly or daily batch processing. This confirms the assumption that the microdynamics of such indicators as the intensity of traffic flow, the frequency of calls to public services, or the level of workload of medical institutions contain important predictive information for intraday forecasting of urban load. Secondly, explicit modeling of deviations from adaptive baselines (EWMA) makes it possible to reliably separate significant structural changes in urban activity from random fluctuations, which increases the stability and reliability of predictive models.

From the point of view of the system architecture, a significant reduction in trend detection latency underscores the importance of streaming analytical solutions for operational urban management tasks. Early detection of emerging changes—such as increasing traffic congestion, a sharp increase in calls to digital government services, or a surge in patients in medical institutions—allows management decisions to be made in advance, including adapting traffic management schemes, reallocating resources in service centers and medical organizations, as well as dynamically configuring digital infrastructure. Unlike existing real-time solutions, which are often limited to descriptive monitoring, the proposed approach combines trend detection, forecasting, and decision support within a single closed control loop.

A comparison with existing studies indicates that the presented work expands on previously proposed approaches in several areas. Although many Smart City studies demonstrate the effectiveness of deep learning methods or the integration of heterogeneous data sources, most of them rely on batch processing and extended time intervals. In contrast, the proposed system is focused on low processing latency and continuous enrichment of models with features, which makes intraday forecasting possible without loss of statistical stability. In addition, the direct integration of trend signals into the forecasting module facilitates the transition from disparate analytical components to an integrated decision support system for a smart city. The performance of the proposed AISSC framework is designed to remain stable under moderate variations in streaming workload, including changes in data volume, event arrival frequency, and stream velocity. The distributed edge–cloud architecture enables computational tasks to be partitioned across processing nodes, while sliding-window aggregation maintains a bounded amount of data for real-time analysis regardless of the cumulative stream size. Furthermore, adaptive EWMA baselines continuously adjust to changing event rates, reducing sensitivity to temporary traffic bursts and natural fluctuations in user activity. Consequently, although increased stream velocity may proportionally increase computational load and resource utilization, the analytical pipeline preserves trend detection reliability and forecasting consistency by combining adaptive normalization, window-based processing, and scalable distributed execution. Enormous streaming rates beyond the available computational resources may require additional hardware scaling or window-size adaptation, which represents an important direction for future deployment studies. Although the proposed AISSC framework is designed to adapt to rapidly changing streaming environments, the current experimental evaluation mainly accounts for normal operational conditions represented by benchmark smart city datasets. Future work will include dedicated stress-testing with synthetic and real emergency scenarios, such as large public gatherings, transportation network failures, natural disasters and large-scale service interruptions to quantitatively evaluate response time, forecasting stability and operational resilience under extreme urban disruptions.

At the same time, the proposed AISSC has limitations. As, the effectiveness of the system largely depends on the correct adjustment of smoothing parameters and thresholds, especially in conditions of high volatility of urban flows and irregular events (accidents, mass events, emergencies). Scaling the solution with a significant increase in the volume of events may require additional computing resources and optimization of the data flow processing infrastructure. As directions for further research, it seems advisable to study methods for adaptive parameter tuning, develop more resource-efficient scaling strategies, and integrate additional external data sources such as weather conditions, urban events, and aggregated signals from open digital platforms.

In general, the results of the discussion confirm that real-time trend analytics are a promising and practically applicable direction for the development of modern smart city systems. A consistent combination of statistical modeling, data streaming, and urban management tasks provides more adaptive and informed management solutions in a dynamic and complex urban environment.

## 7 Conclusion and future work

This study presents an AI-driven real-time trend analytics framework for smart city decision-making. Combining streaming data input, short-window feature engineering, statistically grounded trend detection, and short-term forecasting addresses batch-oriented urban analytics' fundamental shortcomings. The system adapts to urban dynamics to discover behavioral shifts, demand surges, and service-level abnormalities, unlike traditional methods that use historical aggregation. The recommended method improves analytical responsiveness and forecasting accuracy in genuine smart city circumstances. The system's low trend detection latency and high forecast update frequency demonstrate real-time urban management. MAPE and WMAPE gains show that trend-based signals and service-level aggregation work in predictive models. These results show that adaptive statistical baselines and AI-driven features improve transportation, digital public services, and healthcare operational planning.

The proposed layered architecture is scalable, modular, and interoperable, making it suited for various urban infrastructures and data ecosystems. It also improves numerical performance. A reinforcement learning module lets city services adjust regulations and resource distribution depending on observed patterns. This approach provides analytical insights and a platform for data-driven urban governance. Despite these advances, research challenges and opportunities persist. Window sizes, smoothing factors, and detection thresholds affect framework performance, especially in volatile or rare-event scenarios. Fixed parameters worked in the studied circumstances, however meta-learning or self-optimizing control mechanisms may increase robustness across urban situations. Weather, large-scale public events, and social media signals will be added to improve forecast accuracy and situational awareness. Advanced deep learning architectures like temporal graph neural networks or hybrid transformer-based models can improve urban service spatial–temporal dependency analysis. Finally, system stability, processing efficiency, and policy impact require large-scale real-world deployments and longitudinal studies. This study shows that real-time AI-driven trend analytics can manage complex smart city systems. The proposed architecture connects streaming data, statistical dependability, and actionable intelligence to create flexible, resilient, and data-driven cities.

## Data Availability

The raw data supporting the conclusions of this article will be made available by the authors, without undue reservation.

## Appendix

**Table A1:** Symbols and their description.

Symbols	Description
θ^+^, θ^−^	Trend detection thresholds
δ	Stability threshold
η	Informativeness threshold
ϵ	Numerical stability constant
